# Beyond Conversion Chemistry: Unlocking a Cooperative Solid-Solution–Capacitive Sodium-Storage Mechanism in Nickel Phosphide

**DOI:** 10.1007/s40820-026-02076-0

**Published:** 2026-02-13

**Authors:** Jiaqin Liu, Tongzhen Wang, Jie Yang, Yulei Li, Zhaoqian Li, Jiewu Cui, Yan Yu, Yucheng Wu

**Affiliations:** 1 State Key Laboratory of Chemical Resource Engineering, College of Chemistry, Beijing University of Chemical Technology, Beijing, 100029 People’s Republic of China; 2 School of Mechanical Engineering, School of Materials Science and Engineering, Engineering Research Center of Advanced Composite Materials Design & Application of Anhui Province, Hefei University of Technology, Hefei, 230009 People’s Republic of China; 3 School of New Energy Engineering, Hefei Institute of Technology, Hefei, 238706 People’s Republic of China; 4 Department of Materials Science and Engineering, CAS Key Laboratory of Materials for Energy Conversion, Hefei National Research Center for Physical Sciences at the Microscale, University of Science and Technology of China, Hefei, 230026 People’s Republic of China

**Keywords:** Sodium-ion battery, Anode, Ni_2_P, Solid-solution, Pseudocapacitive

## Abstract

Nickel-rich nickel phosphide (Ni_2_P) has emerged as a promising sodium-ion battery anode owing to its high theoretical capacity and intrinsic electronic conductivity, yet its charge storage chemistry remains controversial and is often oversimplified as a conversion reaction. Herein, we design a freestanding Ni_2_P composite electrode composed of ultrasmall Ni_2_P nanocrystals embedded within a phosphorus-doped, graphene-like porous carbon matrix. Comprehensive in-situ and *ex-situ* analyses unequivocally demonstrate an interstitial solid-solution mechanism, wherein Na^+^ ions reversibly occupy lattice interstitials via (111)-oriented interplanar channels, inducing reversible lattice breathing without phase transformation. This bulk intercalation process is synergistically coupled with a substantial pseudocapacitive contribution, establishing a cooperative dual-mode storage mechanism. Benefiting from this solid-solution–capacitive chemistry, the electrode delivers a high reversible capacity (≈560 mAh g^−1^), outstanding rate capability (135 mAh g^−1^ at 10 A g^−1^), and exceptional long-term stability (263 mAh g^−1^ after 2000 cycles). When paired with a Na_3_V_2_(PO_4_)_3_@C cathode, the full cell achieves a high-energy density of 245 Wh kg^−1^. This work establishes solid-solution–capacitive coupling as a general paradigm for designing high-rate and durable sodium-ion battery anodes.

**Supplementary Information:**

The online version contains supplementary material available at 10.1007/s40820-026-02076-0.

## Highlights

- A cooperative dual-mode sodium-storage mechanism, combining interstitial solid-solution intercalation and surface pseudocapacitance, is identified, diverging from the conventional conversion-dominated chemistry of nickel phosphides.
- In-situ/*ex-situ* analyses provide direct evidence of reversible Na^+^ insertion into lattice interstitials through (111)-oriented interplanar channels, enabling low-strain lattice breathing without phase transformation.
- The freestanding Ni_2_P composite electrode achieves exceptional performance, including high reversible capacity (≈560 mAh g^−1^), remarkable rate capability (135 mAh g^−1^ at 10 A g^−1^), and long-term stability over 2000 cycles.

**Supplementary Information:**

The online version contains supplementary material available at 10.1007/s40820-026-02076-0.

## Graphical abstract

**Figure:**
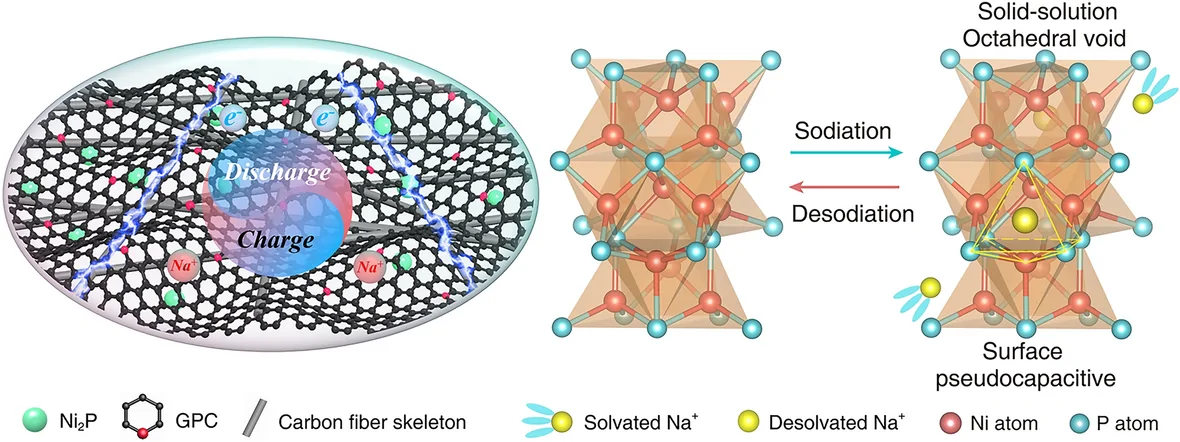

## Introduction

Given the pressing need for cost-effective and sustainable energy storage, sodium-ion batteries (SIBs) have emerged as a compelling post-lithium alternative, driven by the earth-abundance and low cost of sodium, and envisioned to play a pivotal role in stabilizing large-scale renewable energy grids [1–4]. However, the practical deployment of SIBs is hampered by the intrinsic characteristics of Na^+^—its large ionic radius (1.02 Å) and relatively high redox potential (-2.71 V vs. SHE) relative to Li^+^ (-3.04 V vs. SHE) [5, 6]. Such intrinsic features collectively result in sluggish solid-state diffusion kinetics, limited energy density, and pronounced structural instability in host materials during cycling [7–9]. Consequently, developing anode materials with rapid ion/electron transport, robust structural resilience, and high reversible capacity remains a crucial yet challenging pursuit.

Among various anode candidates, nickel-rich nickel phosphide (Ni_2_P) stands out due to its distinctive bonding characteristics arising from the synergistic interplay between metallic Ni–Ni bonding and a robust covalent Ni–P framework [10, 11]. This dual bonding nature endows Ni_2_P with high intrinsic electronic conductivity, structural robustness, and tunable redox properties, thereby positioning it as a promising sodium-ion battery anode, as reflected by its high theoretical capacity and advantageous operating voltages [12–15]. In particular, compared with hard carbon, the current commercial benchmark characterized by limited intrinsic conductivity, moderate capacity, and low tap density, Ni_2_P provides markedly higher electronic conductivity, a substantially higher theoretical capacity, and a greater tap density conducive to enhancing volumetric energy density, underscoring its potential for high-rate and high-energy SIB applications [1, 16, 17]. Despite these advantages, the Na^+^ storage chemistry of Ni_2_P remains controversial. Conventionally, the prevailing model for Na^+^ storage predominantly ascribes to a conversion reaction mechanism, culminating in the formation of Na_3_P and metallic Ni [18–20]. However, recent in-situ and operando characterizations have repeatedly failed to identify these expected crystalline products, revealing a clear inconsistency with the conventional mechanism and highlighting a critical gap in our mechanistic understanding of Na^+^ storage in Ni_2_P [21].

At the nanoscale, however, sodium-storage mechanisms are governed by distinct thermodynamic and kinetic constraints that deviate markedly from bulk behavior [22–24]. When the particle size is reduced below a critical threshold (~ 20 nm), the enlarged surface-to-volume ratio and elevated nucleation barrier suppress classical conversion reactions [25], giving rise instead to an interstitial solid-solution–type process complemented by a surface redox pseudocapacitive contribution [26–29]. This cooperative intercalation–capacitive mechanism has been documented in several transition-metal chalcogenides [30–33]. However, whether a similar mechanism operates in transition-metal phosphides (TMPs) such as Ni_2_P remains unclear, largely owing to the lack of direct in-situ structural or spectroscopic evidence.

In this work, we resolve the long-standing mechanistic ambiguity of Ni_2_P by constructing a hierarchical, freestanding Ni_2_P@GPC/CFP composite electrode, integrating ultrasmall Ni_2_P nanocrystals into a graphene-like phosphorus-doped carbon (GPC) matrix on a carbon fiber paper (CFP) scaffold. This composite electrode serves as both a high-performance anode and a platform for mechanistic exploration. In-situ X-ray diffraction, quasi-insitu X-ray photoelectron spectroscopy, and density functional theory analyses collectively uncover a cooperative sodium-storage mechanism, in which Na^+^ ions reversibly access interstitial sites through (111)-oriented interplanar channels, inducing low-strain lattice breathing without phase transformation. A concurrent pseudocapacitive process further accelerates charge-storage kinetics. This conversion-free, dual-mode solid-solution–capacitive behavior redefines the sodium-storage mechanism in Ni_2_P and establishes a generalizable design principle for high-rate and durable transition-metal phosphide anodes.

## Experimental Section

### Materials

Carbon fiber paper (CFP) was obtained from Cetech Co., Ltd. Nickel acetate (Ni(CH_3_COO)_2_), glucose (C_6_H_12_O_6_), potassium hydroxide (KOH), red phosphorus (P), sulfuric acid (H_2_SO_4_, ≥ 98 wt%), hydrochloric acid (HCl, 35 wt%), nitric acid (HNO_3_, ≥ 65 wt%), oxalic acid dihydrate (C_2_H_2_O_4_·2H_2_O), sodium dihydrogen phosphate dihydrate (NaH_2_PO_4_·2H_2_O), n-propanol (C_3_H_6_OH), sodium (Na) metal, super P, polyvinylidene fluoride (PVDF), and N-methyl-2-pyrrolidone (NMP) were purchased from Sinopharm Chemical Reagent Co., Ltd. (China). Vanadium pentoxide (V_2_O_5_) was obtained from Xiya Chemical Co., Ltd. GF/D glass microfiber membrane, sodium perchlorate (NaClO_4_), ethylene carbonate (EC), dimethyl carbonate (DMC), fluoroethylene carbonate (FEC), and aluminum (Al) foil were purchased from the DoDoChem. Argon (Ar) and Ar/H_2_ (9:1, v/v) were supplied by Hefei Kexun Chemical Co., Ltd.

### Synthesis of Ni_2_P@GPC/CFP Composite

To synthesize the Ni_2_P@GPC/CFP composite, 4 mmol of nickel acetate and 5.5 mmol of glucose were dissolved in 50 mL deionized water under magnetic stirring. Subsequently, 50 mL of 1.5 M KOH solution was added dropwise, followed by continuous stirring at room temperature for 6 h. Then, 6 mmol of red phosphorus was added, and the mixture was stirred for an additional 12 h. The resulting mixture was heated to 80 °C to partially evaporate the solvent, yielding a viscous precursor slurry.

The precursor slurry was uniformly coated onto CFP (areal density: 2.0 mg cm^−2^) and dried under vacuum at 60 °C for 12 h, resulting in an areal precursor mass loading of 3–5 mg cm^−2^. The coated CFP was subsequently annealed at 850 °C for 4 h under an Ar atmosphere at a heating rate of 3 °C min^−1^. The annealed product was sequentially washed with 1 M HCl and deionized water, and then dried under vacuum at 60 °C for 24 h to obtain the final Ni_2_P@GPC/CFP composite. The mass loading of the active Ni_2_P@GPC was 1.4 ± 0.2 mg cm^−2^.

### Synthesis of GPC/CFP and Ni_2_P

The GPC/CFP composite was obtained by selectively removing Ni_2_P from the Ni_2_P@GPC/CFP electrode through aqua regia etching, followed by thorough rinsing with deionized water and drying. For comparison, pure Ni_2_P was synthesized using the same protocol as Ni_2_P@GPC/CFP, except that glucose and the CFP substrate were omitted.

### Synthesis of Na_3_V_2_(PO_4_)_3_@C (NVP@C) Composite

In a typical process, 3.95 mmol of V_2_O_5_ and 12 mmol of C_2_H_2_O_4_·2H_2_O were dissolved in 40 mL of deionized water under stirring at 70 °C for 1 h to form a light-blue solution. Subsequently, 11.8 mmol of NaH_2_PO_4_·2H_2_O and 2.2 mmol of glucose were added and stirred for 30 min. Then, 100 mL of n-propanol was added, followed by stirring for an additional hour. The resulting mixture was dried at 90 °C for 6 h to yield a solid precursor.

The dried precursor was ground into a fine powder and subjected to a two-step thermal treatment under an Ar/H_2_ (9:1, v/v) atmosphere: preheating at 350 °C for 4 h at a heating rate of 2 °C min^−1^, followed by calcination at 800 °C for 6 h at a heating rate of 5 °C min^−1^, yielding the final NVP@C composite as a black powder.

### Materials Characterization

X-ray diffraction (XRD, D/Max-2500 V, Rigaku) with Cu_Kα_ radiation (λ = 0.154056 nm) was used to identify the crystalline phases, with scanning performed over 2θ = 10°-80° at a scan rate of 5° min^−1^. The morphology and microstructure were examined using field-emission scanning electron microscopy (FE-SEM, ZEISS Sigma 300) and field-emission transmission electron microscopy (FE-TEM, Talos F200X G2). Elemental composition and distribution were characterized by energy-dispersive X-ray spectroscopy (EDS, Oxford Instruments, Max50). Surface chemical states were analyzed using X-ray photoelectron spectroscopy (XPS, ESCALAB 250Xi, Thermo Scientific). Raman spectroscopy (LabRAM HR Evolution, Horiba) with a 532-nm excitation laser was used to probe structural features and bonding characteristics.

Thermal stability was evaluated by thermogravimetric analysis (TGA, PerkinElmer) conducted in air from 30 to 800 °C at a heating rate of 10 °C min^−1^. Specific surface area and pore structure were measured by Brunauer–Emmett–Teller (BET) analysis (Autosorb-IQ3).

### Cell Assembly and Electrochemical Measurements

CR2032-type coin cells were assembled in an Ar-filled glovebox to evaluate the electrochemical performance of Ni_2_P@GPC/CFP and GPC/CFP. These materials were directly used as working electrodes (12 mm in diameter), with sodium metal as the counter electrode, GF/D glass microfiber membrane as the separator, and 1 M NaClO_4_ in EC/DMC (1:1, v/v) containing 5 wt% FEC as the electrolyte. The amount of electrolyte was controlled at 180 μL per cell. Galvanostatic cycling was performed within 0.01–3.0 V (vs. Na^+^/Na).

In-situ XRD measurements were carried out using a specially designed coin-cell configuration. The in-situ cell was assembled following the same procedure as the half-cell, except that the stainless-steel cathode cap was replaced by a Be window to allow X-ray penetration, whereas the anode cap was replaced by a sealed steel sleeve. The assembled cell was mounted onto the XRD stage with the Be window facing the X-ray beam, and electrical leads were connected to an electrochemical workstation. During the measurement, XRD patterns were continuously collected over 2θ = 30°-60° at a scan rate of 5° min^−1^, while the cell was cycled at 50 mA g^−1^ within 0.01–3.0 V (vs. Na^+^/Na).

For the preparation of Ni_2_P electrodes, a slurry of Ni_2_P, Super P, and polyvinylidene fluoride (PVDF) (8:1:1, w/w) in N-methyl-2-pyrrolidone (NMP) was cast onto Al foil and dried under vacuum at 60 °C for 12 h. Circular disks (12 mm diameter) with an active-material mass loading of 1.4–1.6 mg cm^−2^ were punched and assembled into half-cells under the same conditions described above.

For full-cell testing, the NVP@C cathode was prepared following the same casting procedure. The areal mass loadings of the cathode and anode were controlled at a ratio of 2:1, with the cathode loading fixed at 3.0 mg cm^−2^. Prior to full-cell assembly, the Ni_2_P@GPC/CFP anode was activated at 50 mA g^−1^ for 5 cycles within 0.01–3 V (vs. Na^+^/Na). Full cells were assembled using Ni_2_P@GPC/CFP as the anode and NVP@C as the cathode, and cycled within 0.5–3.7 V. The energy and power densities were calculated using the following equations [34, 35]:

1 $$ E = \frac{I \Delta t \Delta V}{{3.6 m}}\quad ({\text{Wh kg}}^{{ - {1}}} ) $$

2 $$ P = \frac{3600 E}{{\Delta t}}\quad ({\text{W kg}}^{{ - {1}}} ) $$

where *I* (A) is the discharge current, Δ*t* (s) is the discharge time, Δ*V * (V) is the average voltage, and *m* (g) is the total mass of active materials on both electrodes.

Galvanostatic charge–discharge (GCD) and galvanostatic intermittent titration technique (GITT) tests were conducted on a Neware battery testing system. Cyclic voltammetry (CV) and electrochemical impedance spectroscopy (EIS) measurements were performed on an Autolab PGSTAT302N electrochemical workstation. EIS was carried out over a frequency range of 10 mHz to 100 kHz with an amplitude of 5 mV. CV scans were recorded within 0.01–3.0 V at a scan rate of 0.1 mV s^−1^. GITT was applied to estimate the Na^+^ diffusion coefficient. The half-cells were discharged within 0.01–3 V at 50 mA g^−1^ for a 20 min current pulse, followed by a 2-h relaxation period. This pulse-rest sequence was repeated until the cutoff voltage was reached. For EIS analysis, the Warburg coefficient (${\upsigma }_{\mathrm{w}}$) was extracted from the linear relationship between the real part of impedance (*Z′*) and ${\upomega }^{-0.5}$ in the low-frequency region (ω = 2πf). The Na^+^ diffusion coefficient (${D}_{{\mathrm{Na}}^{+}}$) was calculated using the following equations [36]:

3 $$ Z^{\prime}  = R_{e}  + R_{{ct}}  + \sigma _{w} \omega ^{{ - 0.5}}  $$

4 $${D}_{{Na}^{+}}={R}^{2}{T}^{2}/2{A}^{2}{n}^{4}{F}^{4}{C}^{2}{\sigma }_{w}^{2}$$

where *R* is the gas constant, *T* is the absolute temperature, *A* is the electrode surface area, *n* is the number of electrons, *F *is the Faraday constant, and *C* is the molar concentration of Na^+^ in the electrolyte. All electrochemical measurements were carried out at an ambient temperature of 25 °C.

### Theoretical Calculations

Density functional theory (DFT) calculations were performed using the Vienna *Ab* initio Simulation Package (VASP 5.4) [37, 38]. The Perdew–Burke–Ernzerhof (PBE) functional within the generalized gradient approximation (GGA) was employed to describe electron exchange-correlation interactions, and spin polarization was included throughout the calculations [39].

A plane-wave cutoff energy of 550 eV was used. Brillouin-zone sampling was performed using a Monkhorst–Pack *k*-point mesh of 3 × 3 × 1. The convergence criteria were set to 10^–5^ eV for the total energy and 0.03 eV Å^−1^ for the atomic forces. Surface models of graphene-like carbon (GC), graphene-like phosphorus-doped carbon (GPC), and Ni_2_P(111) were constructed using a slab geometry, in which the bottom layers were fixed, while the remaining layers were fully relaxed. A vacuum layer of 10 Å was applied along the z-axis to avoid interactions between adjacent periodic images.

The adsorption energies (E_ad_) of a Na atom on the GC (001), GPC (001), and Ni_2_P (111) surfaces were calculated using:

5 $${E}_{ad}={E}_{Na@Slab}-({E}_{Slab}+1/2{E}_{Na})$$

where ${E}_{Na@Slab}$ is the total energy of a Na atom adsorbed on the surface, ${E}_{Slab}$ is the total energy of the pristine surface, and ${E}_{Na}$ is the energy of bulk metallic sodium per unit cell (containing two Na atoms).

## Results and Discussion

### Microstructure and Structural Characterization

**Fig. 1:**
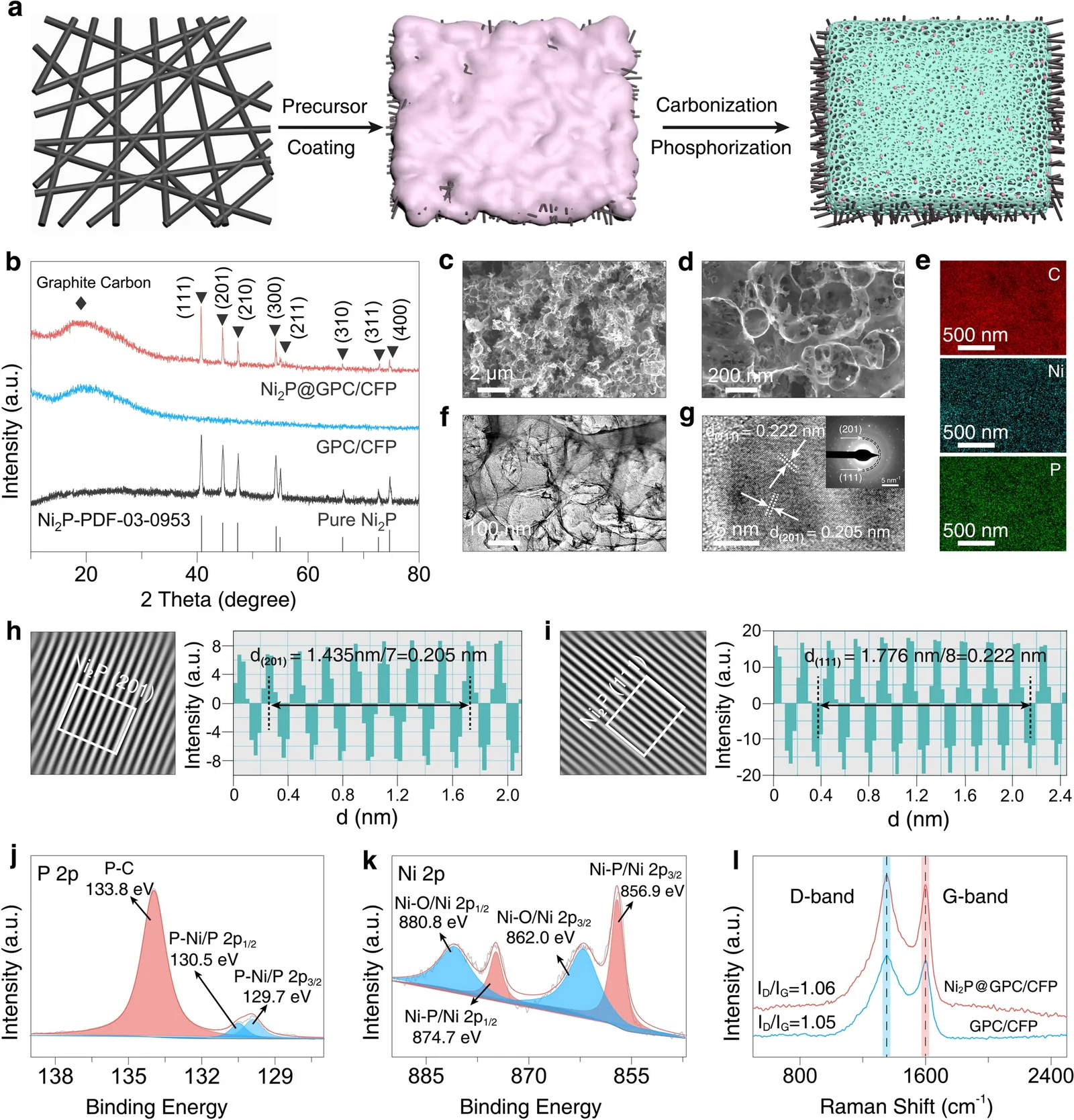
**a** Schematic illustration of the synthesis for Ni_2_P@GPC/CFP. **b** XRD patterns of Ni_2_P@GPC/CFP, GPC/CFP, and pure Ni_2_P. **c, d** SEM images and **e** corresponding EDX elemental mapping of Ni_2_P@GPC/CFP matched with **d**. **f** TEM and **g** HRTEM images of Ni_2_P@GPC/CFP (inset: SAED pattern). **h, i** FFT of Ni_2_P. **j** P 2*p* and **k** Ni 2*p* XPS spectra of Ni_2_P@GPC/CFP. **l** Raman spectra of Ni_2_P@GPC/CFP and GPC/CFP

The Ni_2_P@GPC/CFP composite was synthesized via a synchronous calcination–phosphorization strategy, during which nickel acetate was partially reduced to metallic Ni to catalyze the graphitization of the carbon precursor [40], while red phosphorus served both as a phosphorus source and a reactive modulating agent to tailor the carbon framework [41, 42]. Meanwhile, KOH activation generated abundant hierarchical porosity [43, 44], ultimately yielding a graphene-like, phosphorus-doped carbon network intimately integrated with Ni_2_P nanocrystals (Fig. 1a). X-ray diffraction (XRD) patterns (Fig. 1b) verify the successful formation of crystalline Ni_2_P together with a characteristic graphitic (002) reflection [45], with sharp peaks indexed to the (111), (201), (210), (300), (211), (310), (311), and (400) planes of Ni_2_P (PDF 03-0953). After aqua regia etching, only the carbon (002) peak remains, unambiguously confirming that the Ni_2_P phase is embedded within and stabilized by the porous carbon matrix. Scanning electron microscopy (SEM) images (Figs. 1c, d, and S1, S2) show that the Ni_2_P@GPC uniformly covers the carbon fiber paper (CFP, fiber diameter ~ 10 μm; thickness ~ 230 μm), forming a freestanding three-dimensional conductive network. The CFP scaffold serves simultaneously as current collector and mechanical backbone, eliminating the need for polymer binders, conductive additives, and metal foils. This binder-free architecture simplifies electrode fabrication and significantly enhances volumetric energy density by minimizing inactive components and improving the utilization of electroactive materials. Remarkably, the Ni_2_P@GPC/CFP composite maintains excellent mechanical integrity even under a 500-g load (Fig. S3), demonstrating its robustness for direct use as a SIB anode. Energy-dispersive X-ray spectroscopy (EDX) mapping (Figs. 1e and S4) demonstrates the homogeneous distribution of Ni and P within the graphene-like carbon matrix. Transmission electron microscopy (TEM) (Figs. 1f and S5, S6) further reveals an ultrathin, graphene-like porous carbon architecture that provides fast electron/ion transport pathways while offering nanoscale confinement for Ni_2_P domains, which is critical for accommodating volume fluctuations during cycling. High-resolution TEM (HRTEM) images and selected area electron diffraction (SAED) patterns (Fig. 1g-i) display well-defined lattice fringes corresponding to the Ni_2_P (111) and (201) planes [46], consistent with the XRD results and confirming the high crystallinity of the embedded Ni_2_P nanocrystals.

X-ray photoelectron spectroscopy (XPS) further elucidates the chemical environment of the composite (Fig. S7a). The C 1*s* spectrum displays characteristic peaks assigned to C–C/C = C (284.7 eV), C-O/C-P (285.5 eV), and C =O (288.4 eV) [47–50] (Fig. S7b). The P 2*p* spectrum shows P-Ni (130.5 and 129.7 eV) and P–C (133.8 eV) signals, confirming both the formation of Ni_2_P and P doping in the GPC [51, 52] (Fig. 1j). The Ni 2*p* spectrum exhibits Ni^2+^ features at 856.9 and 874.7 eV together with pronounced satellite peaks (Fig. 1k), indicative of surface oxidation and interfacial Ni–P–C interactions, which may facilitate interfacial charge transfer [53]. Raman spectra reveal distinct D and G bands at 1354 and 1590 cm^−1^ with nearly identical I_D_/I_G_ ratios (1.06 vs. 1.05) for Ni_2_P@GPC/CFP and GPC/CFP, suggesting comparable defect densities and increased surface reactivity resulting from P doping [54] (Fig. 1l). Thermogravimetric analysis (TGA) shows a major weight loss between 140 and 430 °C due to the carbon combustion and Ni_2_P decomposition, followed by the formation of Ni_3_(PO_4_)_2_, Ni_2_P_2_O_7_, and NiO at temperatures above 585 °C [55]. Based on the residual mass, the Ni_2_P content is estimated to be ~ 43.9 wt% (Fig. S7c). Nitrogen adsorption–desorption isotherms exhibit typical type-IV profiles with BET surface area of 221.0 m^2^ g^−1^ and pore volume of 0.412 cm^3^ g^−1^ for Ni_2_P@GPC/CFP, slightly lower than that of GPC/CFP owing to the incorporation of Ni_2_P nanoparticles (Fig. S7d–f). The resulting hierarchical porosity ensures efficient electrolyte infiltration and ion diffusion, thereby supporting fast electrochemical kinetics.

### Electrochemical Performance Evaluation

**Fig. 2:**
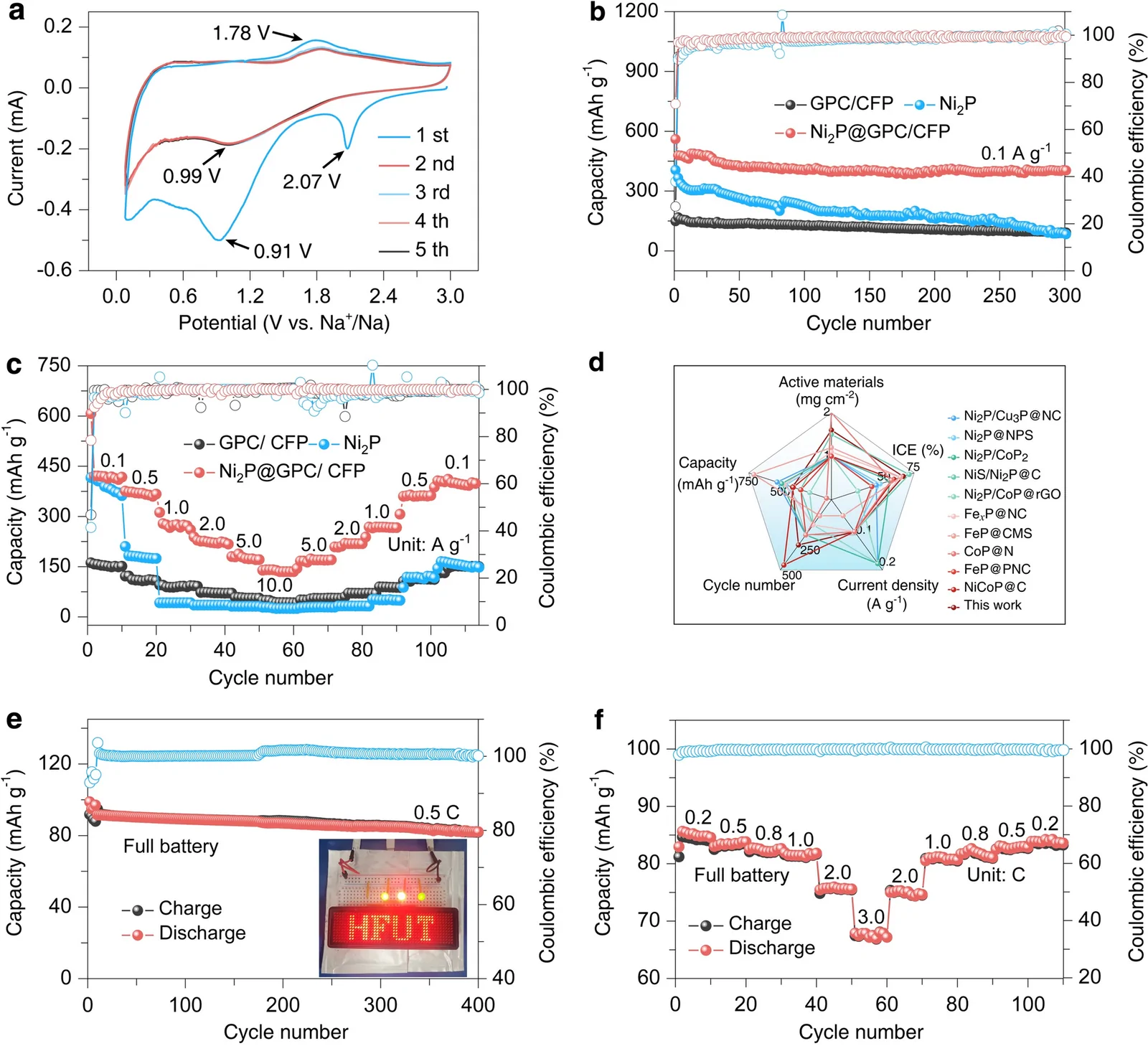
**a** CV curves of Ni_2_P@GPC/CFP at 0.1 mV s^−1^ for the first five cycles. **b** Cycling performance of Ni_2_P@GPC/CFP, GPC/CFP, and Ni_2_P at 100 mA g^−1^. **c** Rate performance of Ni_2_P@GPC/CFP, GPC/CFP, and Ni_2_P. **d** Performance comparison of the Ni_2_P@PC/CFP anode with previously reported transition-metal-phosphide anodes. **e** Cycling performance and **f** rate capability of Ni_2_P@GPC/CFP||NVP@C full cell

The Na^+^ storage performance of Ni_2_P@GPC/CFP was first evaluated by CV and GCD analyses. As shown in Fig. 2a, the CV profiles at 0.1 mV s^−1^ display a broad irreversible cathodic peak at ≈2.07 V in the first scan, associated with SEI formation and electrolyte degradation [56, 57]. The cathodic peak at ≈0.91 V shifts positively to ≈0.99 V in subsequent cycles, indicative of electrode activation with reduced polarization and improved Na^+^ insertion kinetics [47, 58]. A distinct anodic peak at 1.78 V is associated with Na^+^ extraction, and the highly overlapped profiles from the 2nd to 5th cycles confirm excellent reversibility. GCD measurements at 0.1 A g^−1^ show first-cycle discharge/charge capacities of 791/560 mAh g^−1^, giving an initial Coulombic efficiency (ICE) of 70.8% (Fig. 2b). The relatively low ICE is attributed to irreversible Na^+^ consumption during SEI formation and ion trapping [59]. Upon prolonged cycling, the capacity stabilizes at ≈405 mAh g^−1^ after 300 cycles, with a Coulombic efficiency (CE) of ≈99.8% and a remarkably low decay rate of ≈0.09% per cycle. The nearly identical GCD profiles (Fig. S8a) corroborate the structural integrity of the electrode. In contrast, control electrodes exhibit substantially inferior performance: GPC/CFP delivers only 148 mAh g^−1^ initially and fades to 93 mAh g^−1^ after 300 cycles, while bare Ni_2_P undergoes rapid capacity loss due to severe pulverization and electrical disconnection (Figs. 2b and S8c, e).

At high current density (1 A g^−1^), Ni_2_P@GPC/CFP retains 263 mAh g^−1^ after 2000 cycles (78.2% retention, decay ≈ 0.02% per cycle), far outperforming GPC/CFP (79 mAh g^−1^) and Ni_2_P (50 mAh g^−1^) (Fig. S8g). Even at an ultrahigh rate of 10 A g^−1^, it delivers 135 mAh g^−1^, significantly exceeding GPC/CFP and Ni_2_P, and fully recovers to 396 mAh g^−1^ when the current density returns to 0.1 A g^−1^ (Figs. 2c and S8b, d, f), demonstrating outstanding rate capability. Benchmarking against state-of-the-art transition-metal-phosphide anodes (Fig. 2d; Table S1) shows that Ni_2_P@GPC/CFP exhibits competitive overall performance.

To assess practical applicability, a full cell was assembled using Ni_2_P@GPC/CFP as the anode and NVP@C as the cathode. The full cell delivers an initial capacity of 98 mAh g^−1^ at 0.5 C, corresponding to an energy density of 245 Wh kg^−1^ at a power density of 98 W kg^−1^ (based on the total mass of active materials in both electrodes). Remarkably, it retains 82 mAh g^−1^ after 400 cycles at 0.5 C, with a low fading rate of only 0.03% per cycle and a nearly 100% CE (Figs. 2e and S9a). The cell also exhibits robust rate performance, delivering 86 to 68 mAh g^−1^ from 0.2 to 3.0 C, with full recovery to ~ 84 mAh g^−1^ once the current density returns to 0.2 C (Figs.2f and S9b). Even at 2 C, 73 mAh g^−1^ is maintained after 400 cycles (89.3% retention; Fig. S9c, d). The ability of the assembled device to stably power an LED display (inset, Fig. 2e) visually demonstrates its practical applicability.

Ex-situ FE-SEM and EDX analyses after long-term cycling (Figs. S10–S12) reveal a well-preserved porous architecture with uniformly distributed Ni, P, and C, confirming the absence of pulverization or aggregation. This structural robustness stems from the rational material design: hierarchical porosity ensures electrolyte accessibility; the graphene-like carbon scaffold buffers volume fluctuations and maintains electrical conductivity; nanosized Ni_2_P domains shorten diffusion paths and enhance interfacial kinetics; P doping improves electronic/ionic transport and provides additional active sites; and the binder-free configuration maximizes active-material utilization and minimizes interfacial side reactions, thereby offering a clean platform to probe the intrinsic Na-storage mechanism of Ni_2_P.

### Elucidation of Sodium-Storage Kinetics

**Fig. 3:**
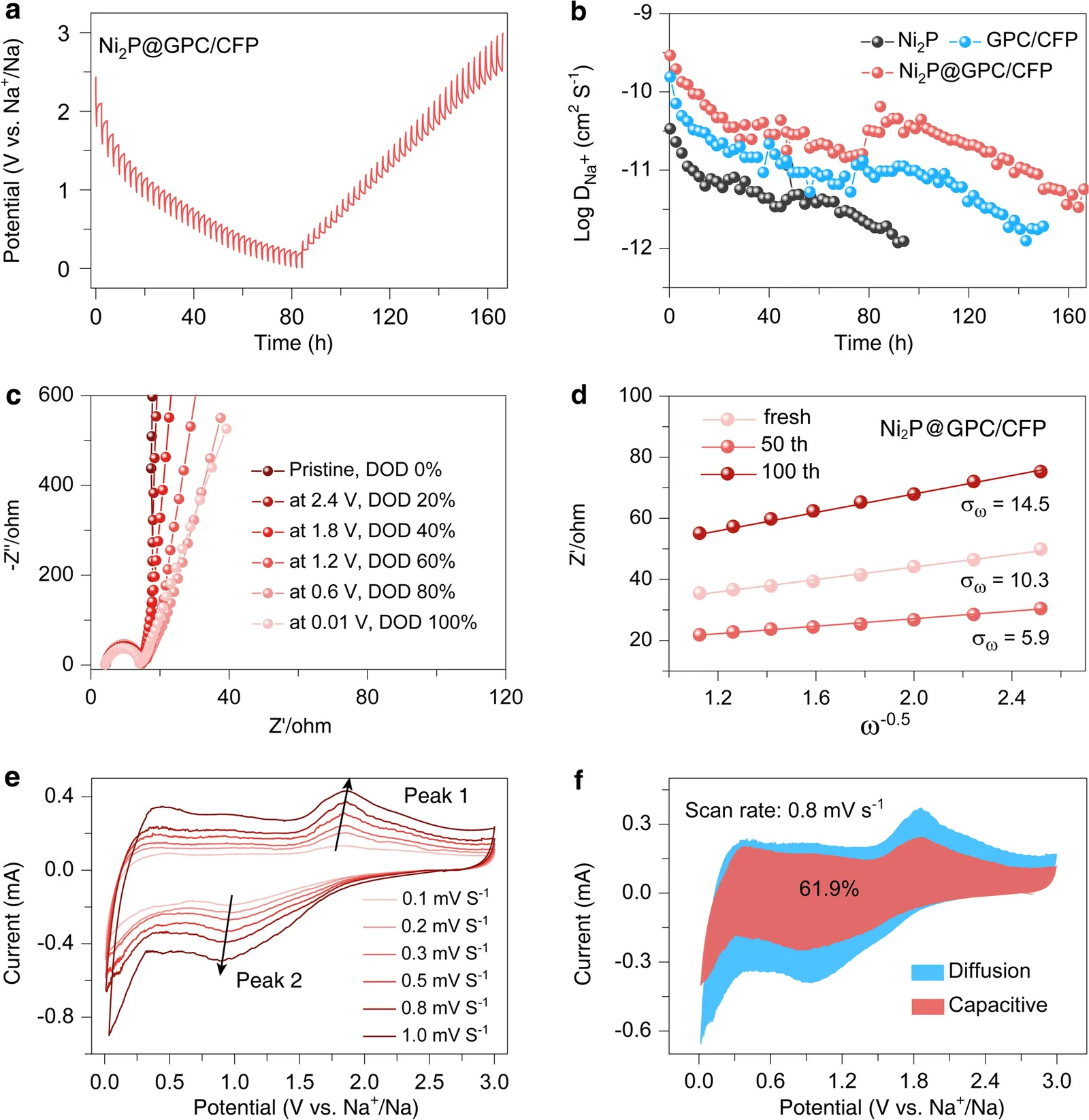
** a** GITT time-potential profiles of Ni_2_P@GPC/CFP. **b** Ion diffusion coefficients of Ni_2_P@GPC/CFP, GPC/CFP, and Ni_2_P. **c** Nyquist plots of Ni_2_P@GPC/CFP at different DOD states (0.01–3.0V). **d** Fitting line of Z′ vs. ω^−0.5^ for EIS before cycling, after 50 and 100 cycles of Ni_2_P@GPC/CFP. **e** CV profiles of Ni_2_P@GPC/CFP at different scan rates, and **f** Capacitive-controlled contribution of Na^+^ at 0.8 mV s^−1^

To gain deeper mechanistic insight, the Na^+^ storage kinetics were investigated using the GITT (Figs. 3a and S13a–c). According to Fick's second law [60], the Na^+^ diffusion coefficient (${D}_{{Na}^{+}}$) can be estimated as: ${D}_{{Na}^{+}}$ = $\frac{4}{\pi \tau }{(\frac{{m}_{B}{V}_{m}}{{M}_{B}S})}^{2}{(\frac{\Delta {E}_{s}}{\Delta {E}_{\tau }})}^{2}$, where *S* (cm^2^) is the effective electrode area; *M*_*B*_ (g mol^−1^), *V*_*m*_ (cm^3^ mol^−1^), and *m*_*B*_ (g) denote the molar mass, molar volume, and mass of the active material, respectively; τ is the pulse duration; ∆*E*_*s*_ (V) is the steady-state voltage change, and ∆*E*_*τ*_ (V) is the transient voltage change during the pulse. The average log(${D}_{{Na}^{+}}$) values of -10.6, -11.0, and -11.3 (Fig. 3b) for Ni_2_P@GPC/CFP, GPC/CFP, and Ni_2_P highlight the enhanced Na^+^ diffusivity in the composite. Nyquist plots at various discharge depths (Fig. 3c) show a consistently low and nearly invariant charge-transfer resistance (~ 10.3 Ω), with minor changes in the Warburg slope, indicative of pseudocapacitive-dominated behavior [32, 61]. For Ni_2_P@GPC/CFP||Na, the interfacial resistance increases modestly after 100 cycles due to gradual SEI growth, yet it remains significantly lower than that of Ni_2_P (Fig. S13d-f; Table S2), indicating that 3D hierarchical GPC framework enhances charge-transfer dynamics and stabilizes the interface during cycling. Furthermore, the Na^+^ diffusion coefficients extracted from the Warburg region of the EIS spectra further corroborate the improved ion transport in Ni_2_P@GPC/CFP. Compared to GPC/CFP and pure Ni_2_P, Ni_2_P@GPC/CFP shows consistently higher Na^+^ diffusion coefficients (Figs. 3d and S13g-h; Table S2), confirming its superior ion transport properties.

To probe the charge-storage mechanism, scan rate-dependent CV analysis of Ni_2_P@GPC/CFP performed (Fig. 3e). The current response (*i*) scales with the scan rate (*v*) according to a power-law (*i* = *av*^*b*^) [62, 63], where a is a variable parameter, and b is an index used to assess the reaction kinetics. The *b* value close to 1.0 typically corresponds to surface capacitive behavior, while *a* value closer to 0.5 indicates a diffusion-dominated process. The *b*-values for oxidation (0.783) and reduction (0.755) suggest a hybrid diffusion-capacitive mechanism that enables fast Na^+^ uptake and release (Fig. S14a). Quantitative deconvolution using *i* = *k*_1_*ν* + *k*_*2*_*ν*^1/2^ reveals that the capacitive contribution dominates at higher scan rates [64, 65], increasing from 48.2% at 0.1 mV s^−1^ to 75.4% at 1.0 mV s^−1^, and reaching 61.9% at 0.8 mV s^−1^ (Figs. 3f and S14b). The predominance of capacitive contributions facilitates fast Na^+^ uptake/release, which underpins the outstanding high-rate capability and structural durability of Ni_2_P@GPC/CFP over prolonged cycling.

### Mechanistic Investigation and Theoretical Validation

**Fig. 4:**
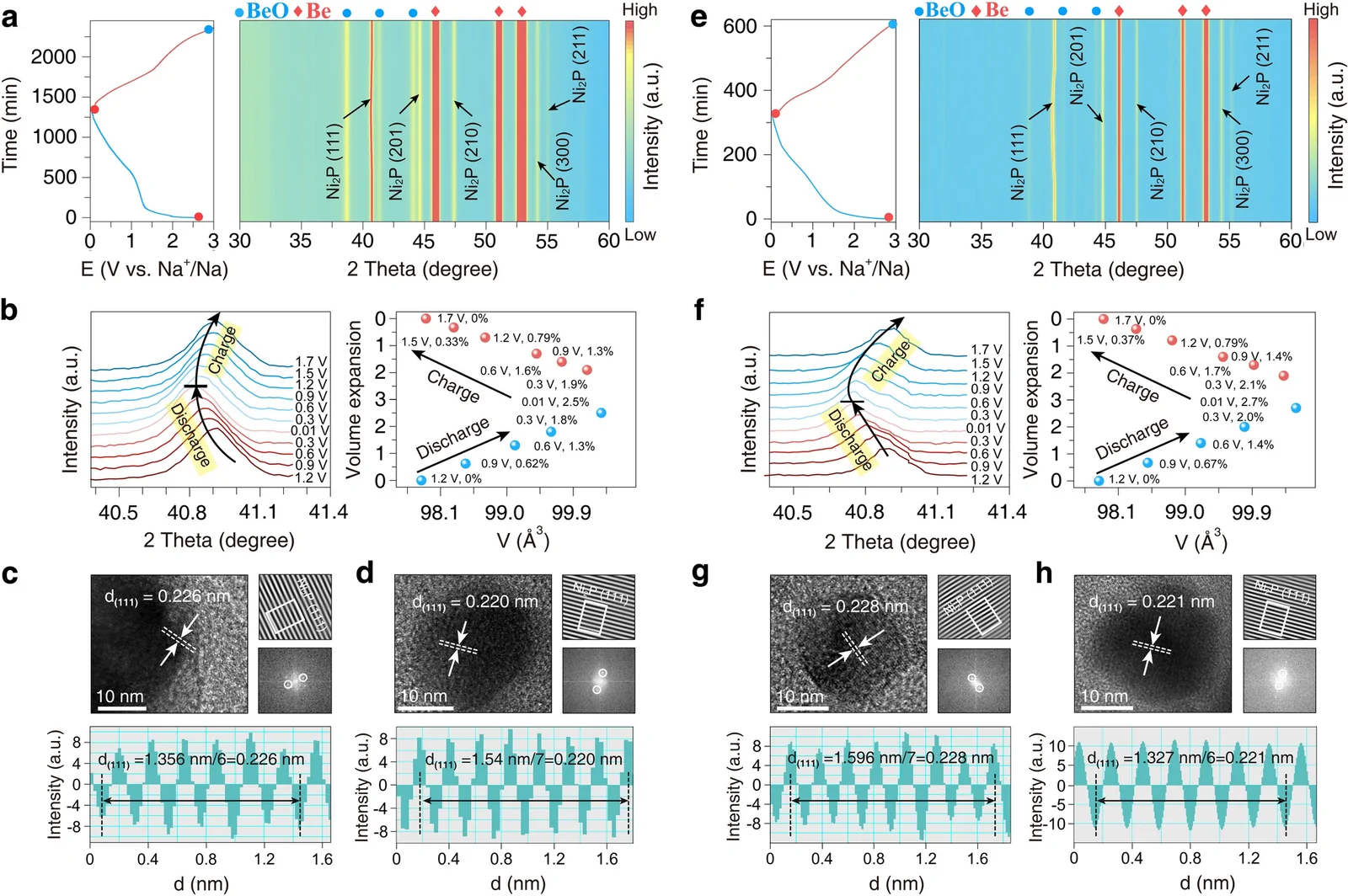
In-situ and ex-situ structure evolution of Ni_2_P@GPC/CFP during sodiation/desodiation. **a** Charge–discharge curve and curve and in-situ XRD patterns at the first cycle. **b** XRD of Ni_2_P (111) and unit cell volume change during the first cycle. **c, d** HRTEM image and corresponding FFT of Ni_2_P at 0.01 V (discharge) and 3.0 V (charge) in the first cycle. **e** Charge–discharge curve and in-situ XRD patterns at the 10th cycle. **f** Ni_2_P (111) evolution and volume change during the 10th cycle. **g, h** HRTEM image and corresponding FFT of Ni_2_P at 0.01 V (discharge) and 3.0 V (charge) in the 10th cycle

The Na-storage mechanism was further elucidated through a combination of in-situ/ex-situ characterizations and DFT calculations. In-situ XRD measurements during both the first and 10th cycles (Fig. 4a, e) reveal that Ni_2_P@GPC/CFP retains its crystalline structure throughout sodiation and desodiation, with no appearance of new peaks, thus excluding a conversion-based process. Notably, the Ni_2_P (111) reflection undergoes a fully reversible shift toward lower 2θ during sodiation and recovers upon desodiation, corresponding to a reversible unit-cell expansion of ~ 2.5% to 2.7% (Fig. 4b, f), indicating the lattice breathing behavior. The interplanar spacing of the Ni_2_P (111) plane (0.220 nm) is significantly larger than the effective size of desolvated Na^+^ (~ 0.102 nm), whereas other low-index planes have substantially smaller spacings. As a result, Na^+^ insertion is geometrically favored along the (111)-oriented interplanar channels, enabling preferential Na^+^ occupation of lattice interstitial sites (e.g., irregular octahedral voids) in Ni_2_P.

*Ex-situ* HRTEM further corroborates this behavior: the (111) lattice spacing expands from ~ 0.220 to ~ 0.226 nm upon discharging to 0.01 V, and contracts back to ~ 0.220 nm after charging to 3.0 V during the first cycle (Fig. 4c, d). A similar trend is observed in the 10th cycle (0.228 → 0.221 nm; Fig. 4g, h). This reversible lattice breathing behavior, sustained across multiple cycles, underscores the low-strain, non-conversion nature of the interstitial solid-solution mechanism in Ni_2_P. Further analysis of in-situ XRD data (Fig. S15) confirms the preservation of the crystallographic integrity of Ni_2_P throughout cycling, evidenced by minimal changes in the intensity ratios of Ni_2_P (201)/BeO (100) and Ni_2_P (210)/BeO (100). Long-term *ex-situ* XRD (Fig. S16) reveals that the (111) peak continues to shift to lower angles during sodiation, but the extent of this shift progressively decreases and stabilizes, suggesting that initial lattice expansion is more pronounced but becomes increasingly accommodated over cycles. Additionally, SEM–EDX mapping at different charge/discharge states after 10 cycles (Figs. S17-S19) shows uniform distributions of Ni, P, and C with no evidence of aggregation or phase separation, further supporting the intercalation-dominated, non-conversion behavior of Na^+^ storage in Ni_2_P.

**Fig. 5:**
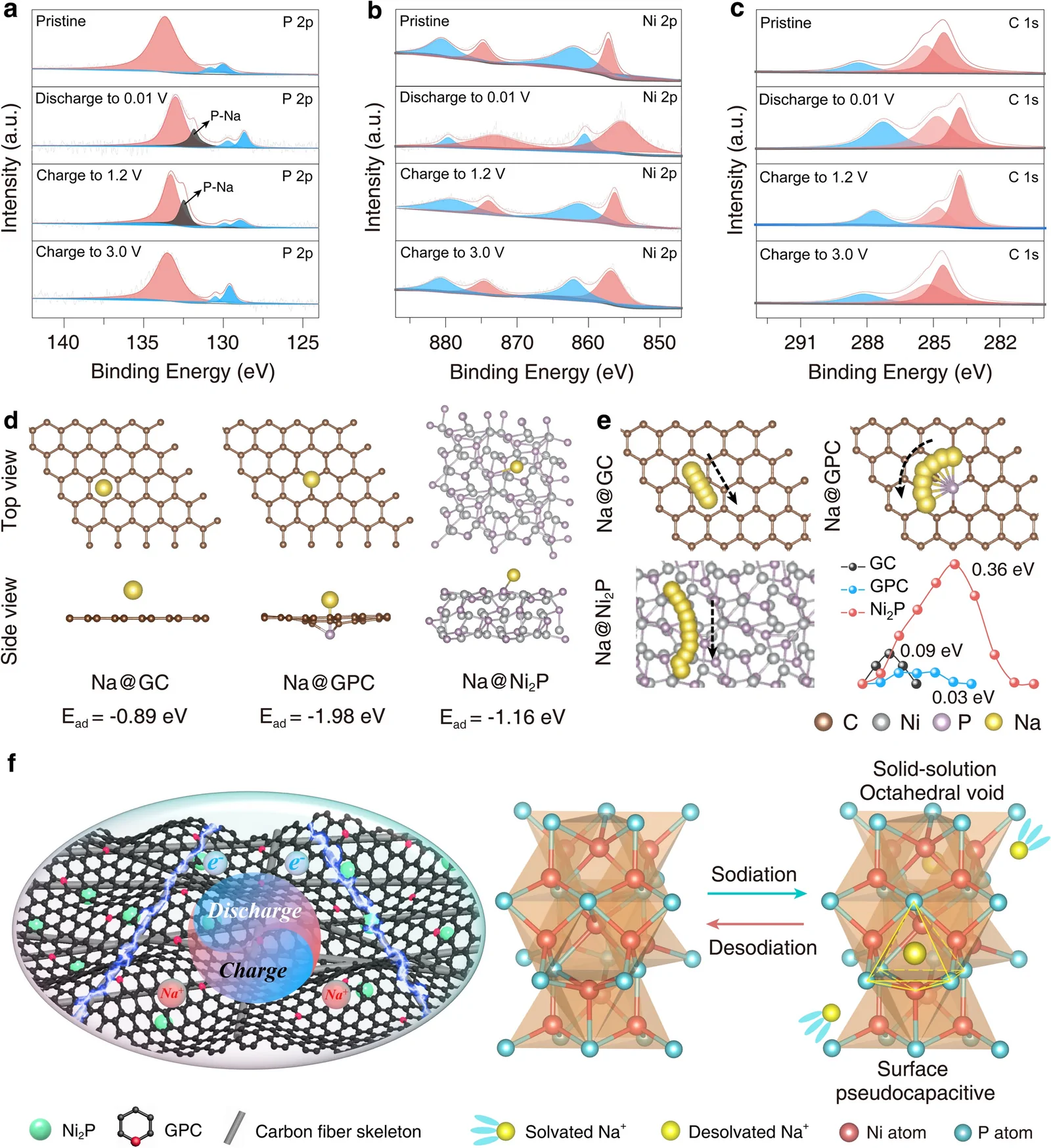
**a–c** Ex-situ XPS analysis of P 2*p*, Ni 2*p* and C 1*s* at different sodiation/desodiation states. **d** Top and front views of Na^+^ adsorption sites on the GC, GPC and Ni_2_P surfaces, along with their corresponding adsorption energies. **e** Na^+^ diffusion paths on GC, GPC and Ni_2_P surfaces, together with the associated diffusion barriers. **f** Schematic illustration of the proposed sodium storage behavior for Ni_2_P@GPC/CFP

Quasi-in-situ XPS further provides insights into the electronic evolution during Na^+^ insertion/extraction. In the P 2*p* spectra (Fig. 5a), pristine Ni_2_P@GPC/CFP displays characteristic P-Ni and P–C peaks. Upon discharging to 0.01 V, a new P-Na feature emerges, accompanied by a negative shift of the P-Ni peak, indicating increased electron density on P as Na^+^ occupies interstitial sites to form Na_*x*_Ni_2_P [66]. During charging, the P-Na signal weakens at 1.2 V and disappears at 3.0 V, with the P-Ni peaks returning to their initial positions, demonstrating excellent reversibility. The Ni 2*p* spectra (Fig. 5b) show parallel negative shifts during sodiation and recovery upon desodiation, confirming reversible electron redistribution around Ni without forming metallic Ni. Meanwhile, the C 1*s* spectra (Fig. 5c) reveal subtle shifts of C–C/C=C and C–O/C–P components upon discharge, which also recover after charging, suggesting a reversible modulation of the carbon scaffold and C-P bonding. These findings collectively demonstrate a fully reversible electronic environment, consistent with an intercalation-dominated solid-solution mechanism.

DFT calculations were also performed to provide atomic-level insights into Na^+^ adsorption and diffusion (Fig. 5d). The calculated adsorption energies are -0.89, -1.98, and -1.16 eV on GC (001), GPC (001), and Ni_2_P (111), respectively, indicating that heteroatom-doped porous carbon offers the most favorable adsorption sites, while Ni_2_P also contributes strong anchoring. The corresponding diffusion barriers are 0.09, 0.03, and 0.36 eV for GC, GPC, and Ni_2_P, respectively (Fig. 5e), suggesting that although Ni_2_P affords strong Na binding, it suffers from sluggish ion transport. Compositing Ni_2_P with conductive GPC therefore combines strong adsorption with ultrafast ion diffusion, reconciling capacity retention with high-rate performance.

Taken together, the structural, spectroscopic, and theoretical findings consistently demonstrate that Na^+^ storage in Ni_2_P@GPC/CFP occurs through an interstitial solid-solution mechanism, in which desolvated Na^+^ ions enter and exit lattice interstitials along the (111) plane pathways without generating Na_3_P or metallic Ni. Figure 5f illustrates the proposed sodium storage behavior for Ni_2_P@GPC/CFP. This mechanism facilitates highly reversible lattice breathing and structural stabilization during cycling. In parallel, kinetics analyses (CV and GITT/EIS) demonstrate dominant pseudocapacitive contributions at higher rates, synergistically complementing the solid-solution process. The dual-mode storage mechanism, comprising solid-solution intercalation coupled with capacitive behavior, underpins the high rate capability, capacity retention, and long-term durability of Ni_2_P@GPC/CFP.

## Conclusions

In summary, we demonstrate a freestanding Ni_2_P-based nanocomposite electrode that enables a dual-mode sodium-storage mechanism, conclusively identified as a cooperative interplay between interstitial solid-solution intercalation and surface pseudocapacitance. This concerted mechanism, which avoids the conventional conversion reaction, is facilitated by the designed hierarchical porosity and nanoconfinement, yielding high reversible specific capacity of ≈405 mAh g^−1^ after 300 cycles, outstanding rate capability (135 mAh g^−1^ at 10 A g^−1^) and long-term stability with a specific capacity of 263 mAh g^−1^ at 1 A g^−1^ after 2000 cycles, corresponding to a capacity retention of 78.2%.

## Supplementary Information

Below is the link to the electronic supplementary material.

Supplementary file1 (DOCX 17027 KB)
