# Supplementary material for: Beyond Conversion Chemistry: Unlocking a Cooperative Solid-Solution–Capacitive Sodium-Storage Mechanism in Nickel Phosphide
Source: Nanomicro Lett. 2026 Feb 13;18:253. doi: 10.1007/s40820-026-02076-0 (PMC12905056; doi:10.1007/s40820-026-02076-0)
Supplement: Supplementary file 1 — Supplementary file1 (DOCX 17027 KB) [file 40820_2026_2076_MOESM1_ESM.docx]

# Supporting Information for

**Beyond Conversion Chemistry: Unlocking a Cooperative Solid-Solution–Capacitive Sodium Storage Mechanism in Nickel Phosphide**

Jiaqin Liu^1,3,*^, Tongzhen Wang^2^, Jie Yang^2^, Yulei Li^2,*^, Zhaoqian Li^3^, Jiewu Cui^2^, Yan Yu^4,*^, Yucheng Wu^2,3,*^

^1^State Key Laboratory of Chemical Resource Engineering, College of Chemistry, Beijing University of Chemical Technology, Beijing 100029, P. R. China

^2^School of Mechanical Engineering, School of Materials Science and Engineering, Engineering Research Center of Advanced Composite Materials Design & Application of Anhui Province, Hefei University of Technology, Hefei 230009, P. R. China

^3^School of New Energy Engineering, Hefei Institute of Technology, Hefei 238706, P. R. China

^4^Hefei National Research Center for Physical Sciences at the Microscale, Department of Materials Science and Engineering, CAS Key Laboratory of Materials for Energy Conversion, University of Science and Technology of China, Hefei 230026, P. R. China

*Corresponding authors. E-mail: [jqliu@buct.edu.cn](mailto:jqliu@buct.edu.cn) (Jiaqin Liu); [lyl@hfut.edu.cn](mailto:lyl@hfut.edu.cn) (Yulei Li); [yanyumse@ustc.edu.cn](mailto:yanyumse@ustc.edu.cn) (Yan Yu); [ycwu@hfut.edu.cn](mailto:ycwu@hfut.edu.cn) (Yucheng Wu)

**Supplementary Figures and Tables**


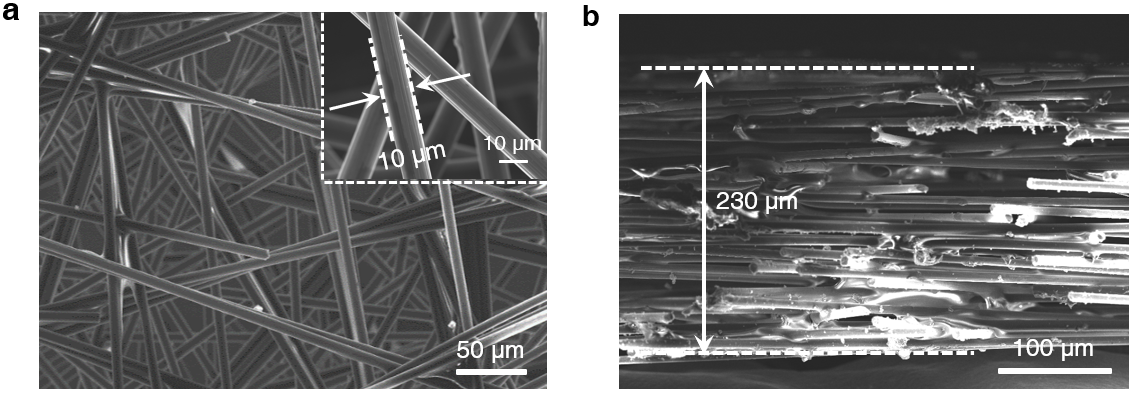


**Fig. S1** **a** Top-view and **b** side-view SEM images of CFP


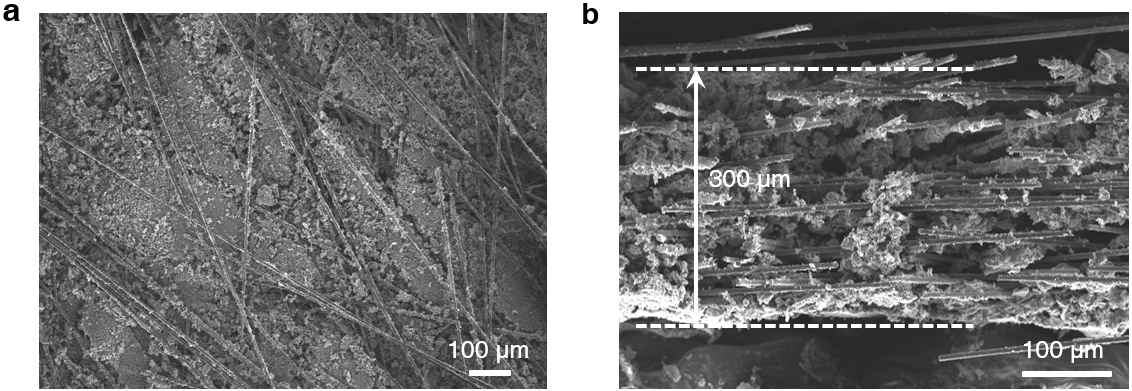


**Fig. S2** **a** Top-view and **b** side-view SEM images of Ni_2_P@GPC/CFP


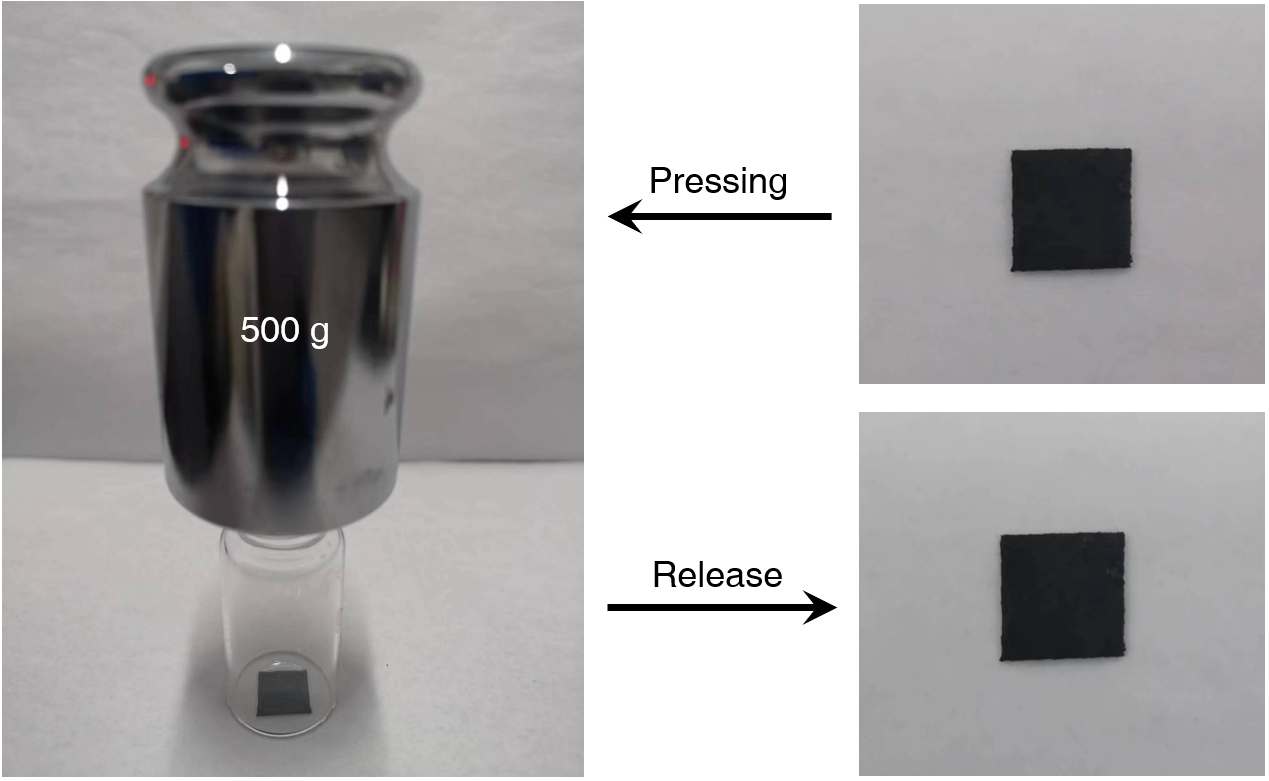


**Fig. S3** Digital photographs showing the mechanical pressing and release test of the Ni_2_P@GPC/CFP


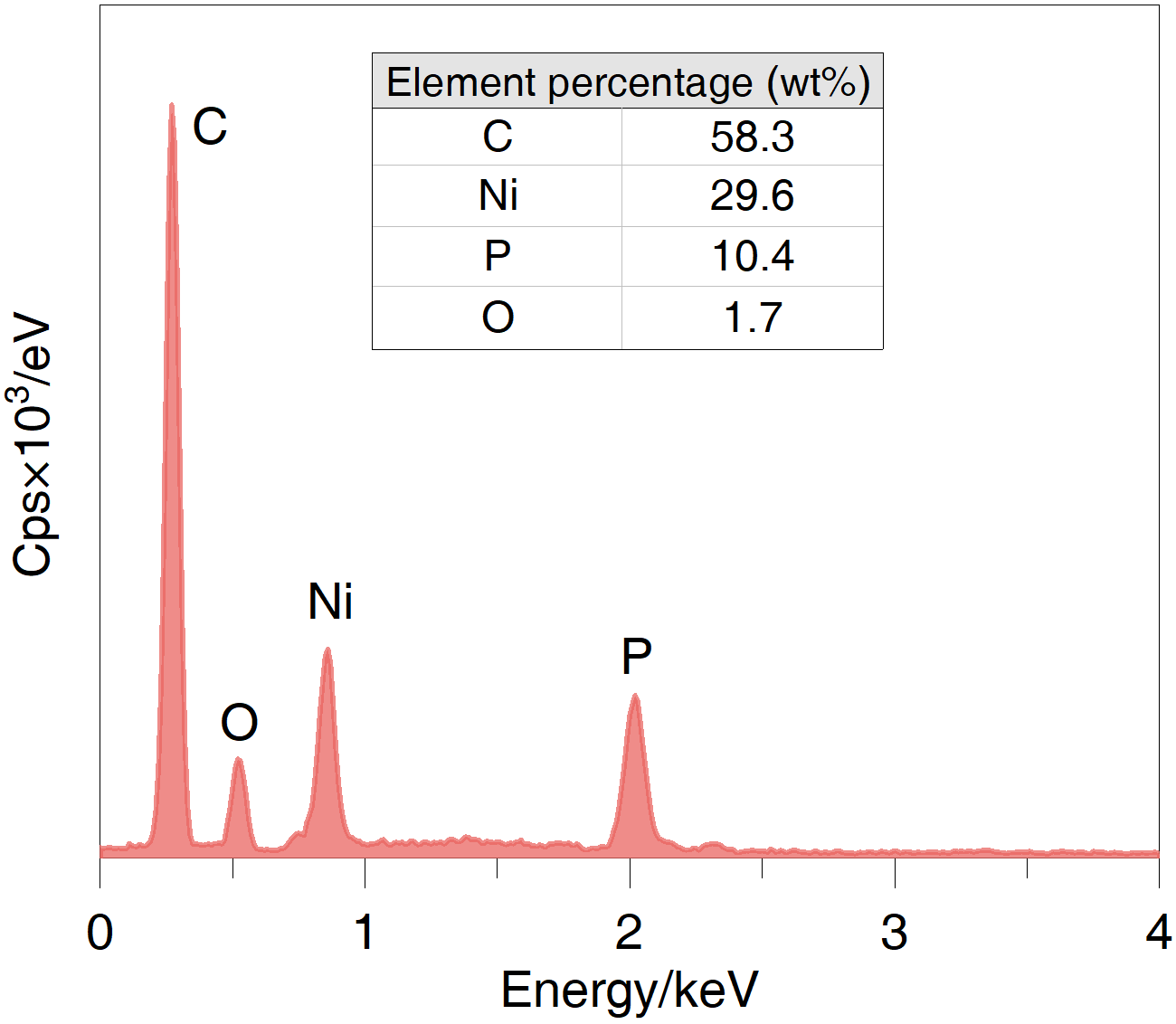


**Fig. S4** EDX spectrum of Ni_2_P@GPC/CFP


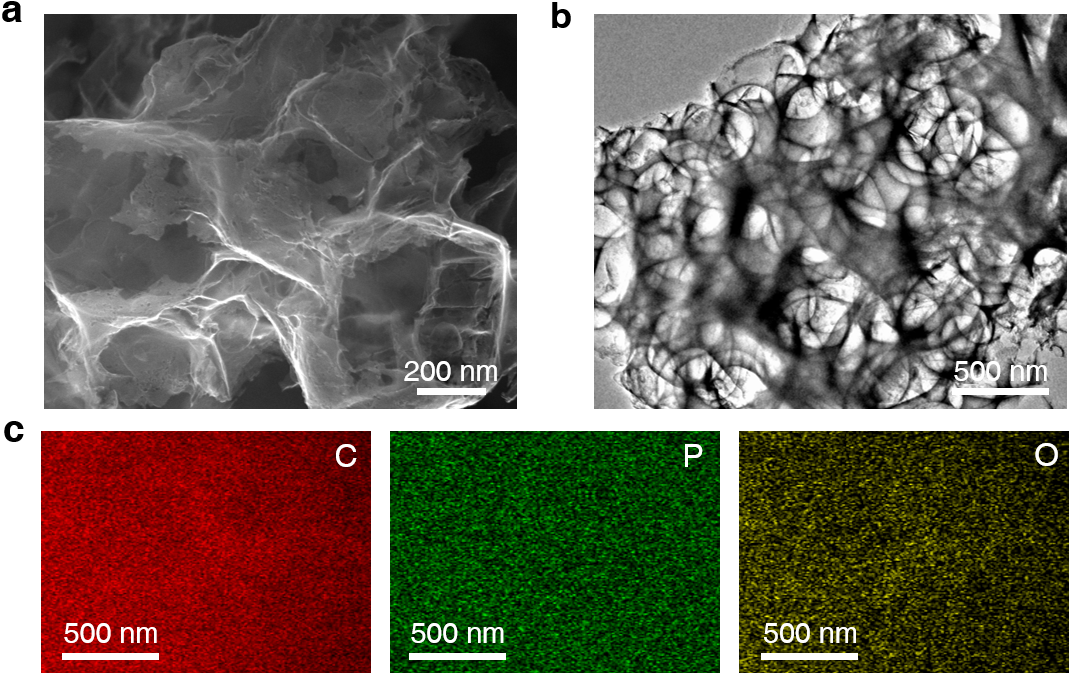


**Fig. S5 a** SEM and **b** TEM images of GPC/CFP. **c** Corresponding EDX elemental mappings of C, P, and O matched with **a**


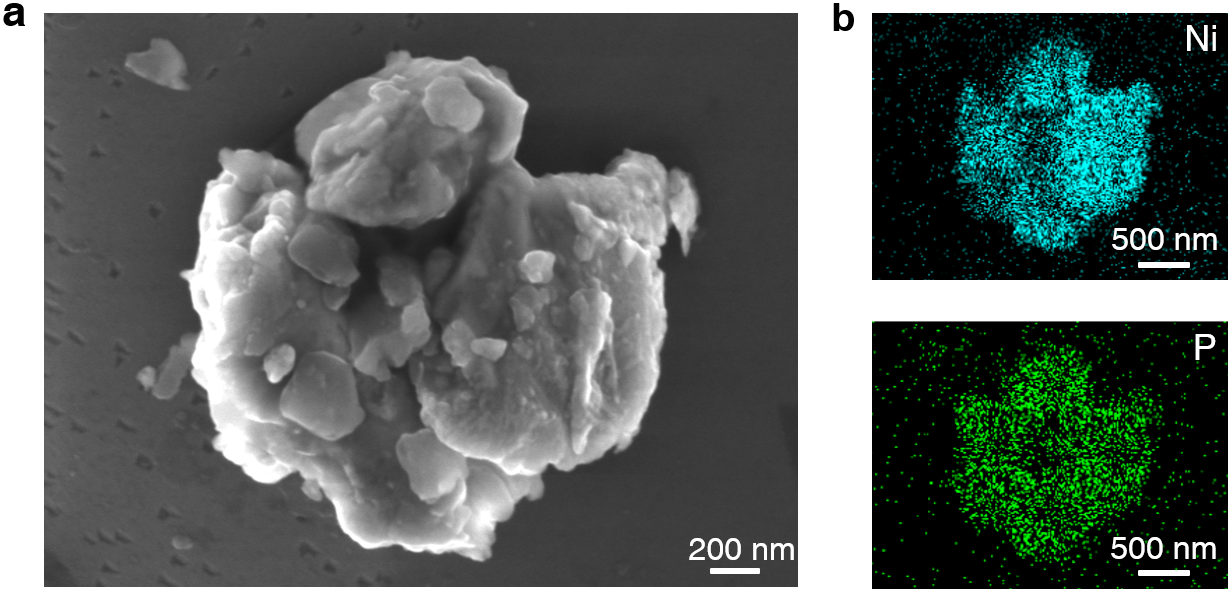


**Fig. S6 a** SEM and **b** corresponding EDX elemental mappings of Ni and P for pure Ni_2_P


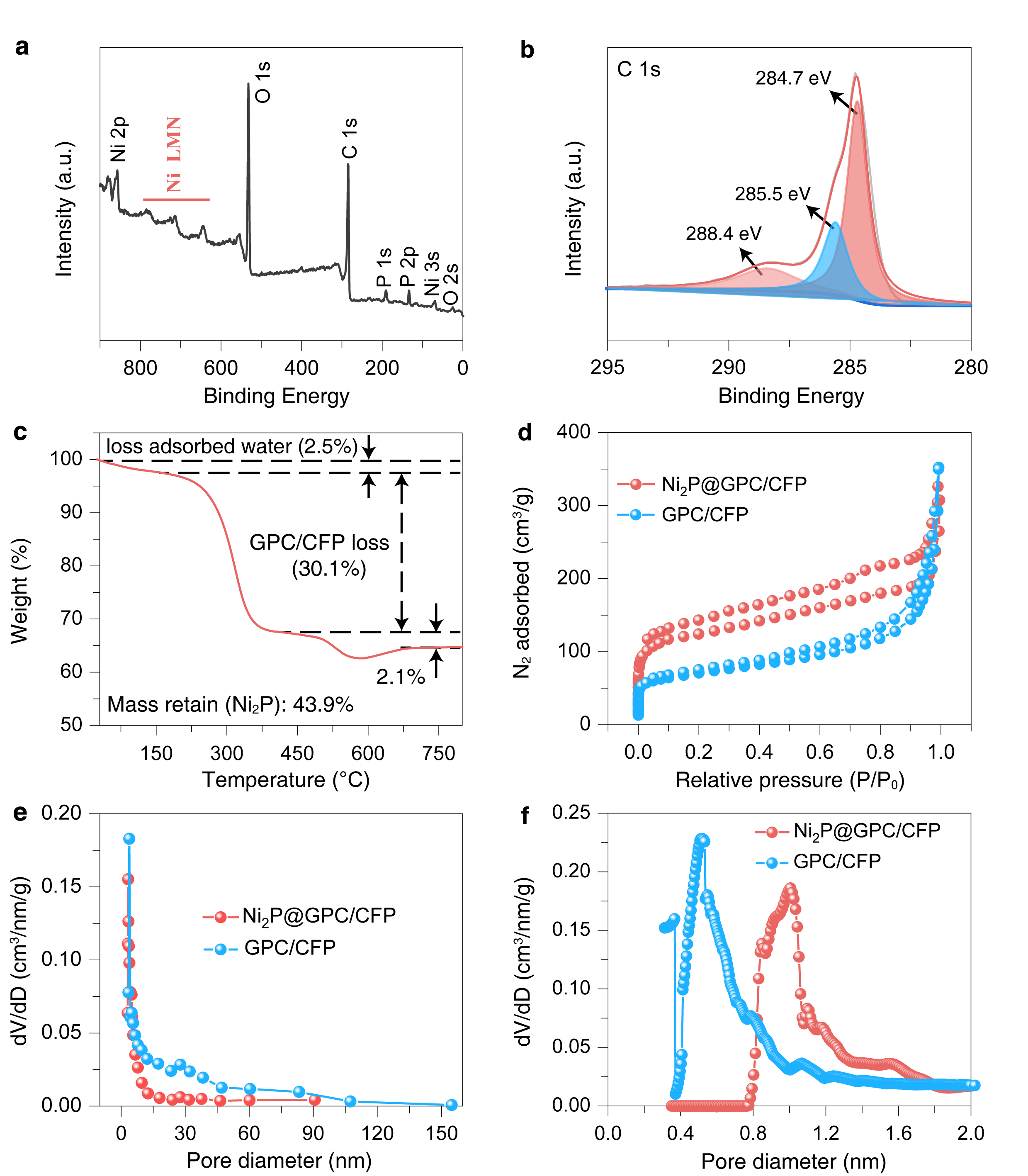


**Fig. S7 a** XPS survey spectrum and **b** C 1s spectrum of Ni_2_P@GPC/CFP. **c** TGA curve. **d** N_2_ adsorption/desorption isotherms. **e** Pore sized distribution and **f** Enlarged pore size distribution below 2 nm of Ni_2_P@GPC/CFP and GPC/CFP


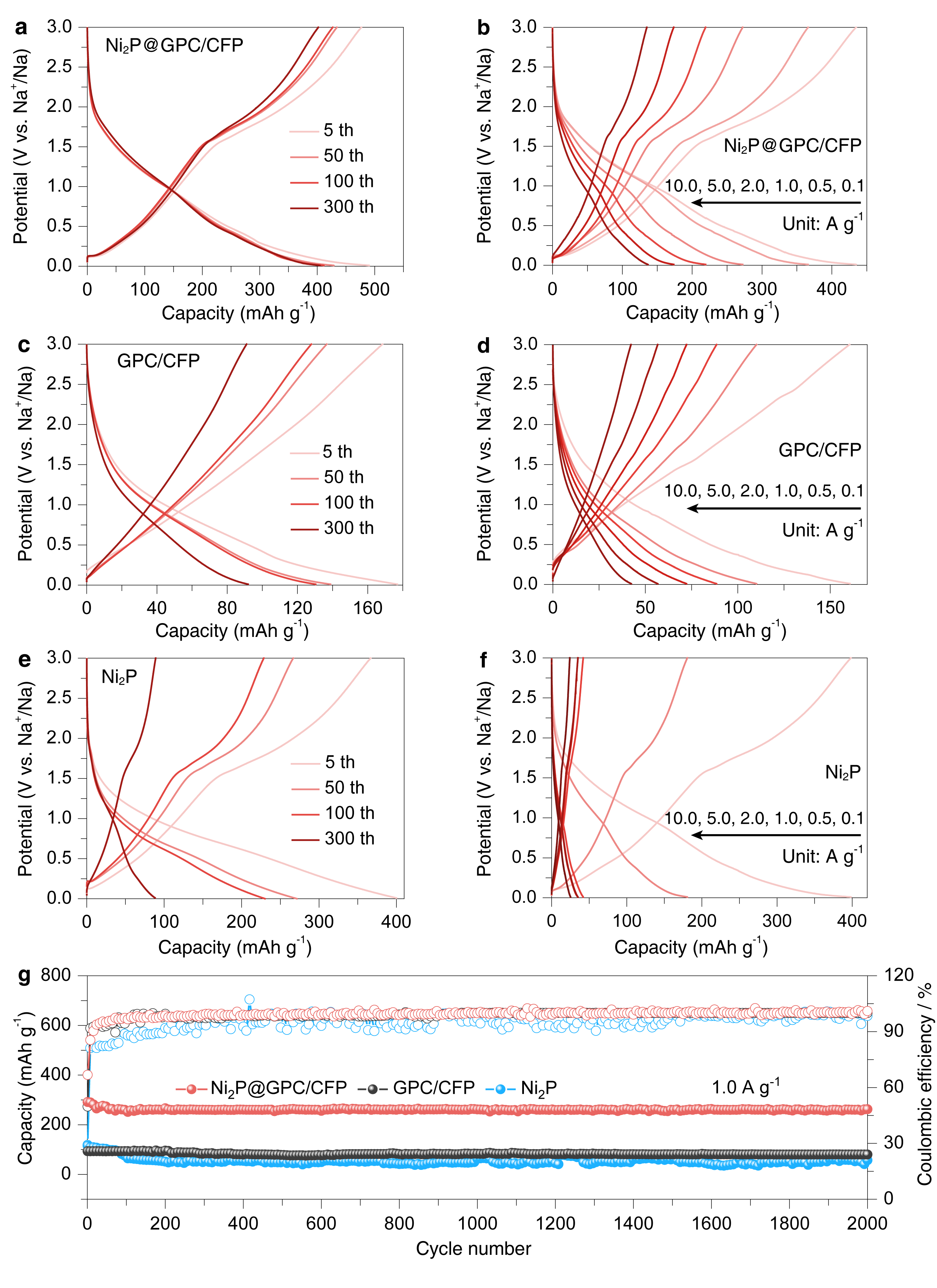


**Fig. S8** **a, c, e** Galvanostatic charge/discharge curves at 100 mA g^-1^ and **b, d, f** rate profiles for Ni_2_P@GPC/CFP, GPC/CFP, and Ni_2_P, respectively. **g** Long-term cycling performance and coulombic efficiency of the three electrodes at 1.0 A^-1^

**Table S1** Performance comparison of the Ni_2_P@PC/CFP anode with previously reported transition-metal-phosphide anodes.

| **Sample** | **Active materials**  **(mg·cm^-2^)** | | **Initial discharge capacities (mAh g^-1^)** | **ICE (%)** | | **Current density (A g ^-1^)** | | **Cycle number** | **Reversible capacity (mAh g^-1^)** | **Rate capability** | **References** |
| --- | --- | --- | --- | --- | --- | --- | --- | --- | --- | --- | --- |
| Ni_2_P@GPC/CFP | | 1.2-1.6 | 921 | 70.8 | 0.1 | | 300 | | 405 | 0.5 A g^-1^/369 mAh g^-1^  1.0 A g^-1^/275 mAh g^-1^  5.0 A g^-1^/174 mAh g^-1^  10.0 A g^-1^/135 mAh g^-1^ | This work |
| Ni_2_P@NPS | | 1.09 | 1570.3 | 51.2 | 0.1 | | 100 | | 457.1 | 0.2 A g^-1^/830 mAh g^-1^  0.8 A g^-1^/644 mAh g^-1^  1.0 A g^-1^/618 mAh g^-1^  2.0 A g^-1^/527 mAh g^-1^ | [S1] |
| Ni_2_P/Cu_3_P@NC | | 0.8-1.2 | 1135 | 47.9 | 0.2 | | 200 | | 526 | 0.2 A g^-1^/568 mAh g^-1^  0.5 A g^-1^/467 mAh g^-1^  1.0 A g^-1^/393 mAh g^-1^  2.0 A g^-1^/334 mAh g^-1^ | [S2] |
| Ni_2_P/CoP@rGO | | 1.0 | 770.1 | 32.4 | 0.2 | | 100 | | 270.4 | 0.1 A g^-1^/332 mAh g^-1^  0.2 A g^-1^/290 mAh g^-1^  0.5 A g^-1^/233 mAh g^-1^  1.0 A g^-1^/197 mAh g^-1^ | [S3] |
| NiS/Ni_2_P@C | | 1.5 | 1295 | 75.8 | 0.1 | | 100 | | 344 | 0.2 A g^-1^/520 mAh g^-1^  0.4 A g^-1^/480 mAh g^-1^  0.8 A g^-1^/410 mAh g^-1^  1.6 A g^-1^/360 mAh g^-1^ | [S4] |
| Ni_2_P/CoP_2_ | | 0.8-1.2 | 876 | 59.5 | 0.2 | | 200 | | 490 | 0.2 A g^-1^/542 mAh g^-1^  0.5 A g^-1^/480 mAh g^-1^  2.0 A g^-1^/421 mAh g^-1^  5.0 A g^-1^/375 mAh g^-1^ | [S5] |
| Fe*_x_*P@NC | | 1.1 | 673.4 | 68.2 | 0.1 | | 100 | | 468.8 | 0.2 A g^-1^/404 mAh g^-1^  0.5 A g^-1^/381 mAh g^-1^  2.0 A g^-1^/336 mAh g^-1^  5.0 A g^-1^/272 mAh g^-1^ | [S6] |
| NiCoP@C | | 1.0 | 1738 | 63 | 0.1 | | 500 | | 400 | 0.2 A g^-1^/676 mAh g^-1^  0.5 A g^-1^/525 mAh g^-1^  0.8 A g^-1^/448 mAh g^-1^  1.0 A g^-1^/385 mAh g^-1^ | [S7] |
| FeP@CMS | | 1.1-1.3 | 1135 | 68.5 | 0.05 | | 10 | | 709 | 0.1 A g^-1^/663 mAh g^-1^  1.0 A g^-1^/443 mAh g^-1^  10.0 A g^-1^/250 mAh g^-1^  20.0 A g^-1^/211 mAh g^-1^ | [S8] |
| FeP@PNC | | 1.0 | 690.4 | 44.2 | 0.1 | | 200 | | 340 | 0.5 A g^-1^/242 mAh g^-1^  1.0 A g^-1^/184 mAh g^-1^  2.0 A g^-1^/121 mAh g^-1^  3.0 A g^-1^/84 mAh g^-1^ | [S9] |
| CoP@N | | 2.0 | 283 | 60.8 | 0.1 | | 300 | | 138 | 0.1 A g^-1^/176 mAh g^-1^  0.5 A g^-1^/130 mAh g^-1^  1.0 A g^-1^/110 mAh g^-1^  2.0 A g^-1^/90 mAh g^-1^ | [S10] |


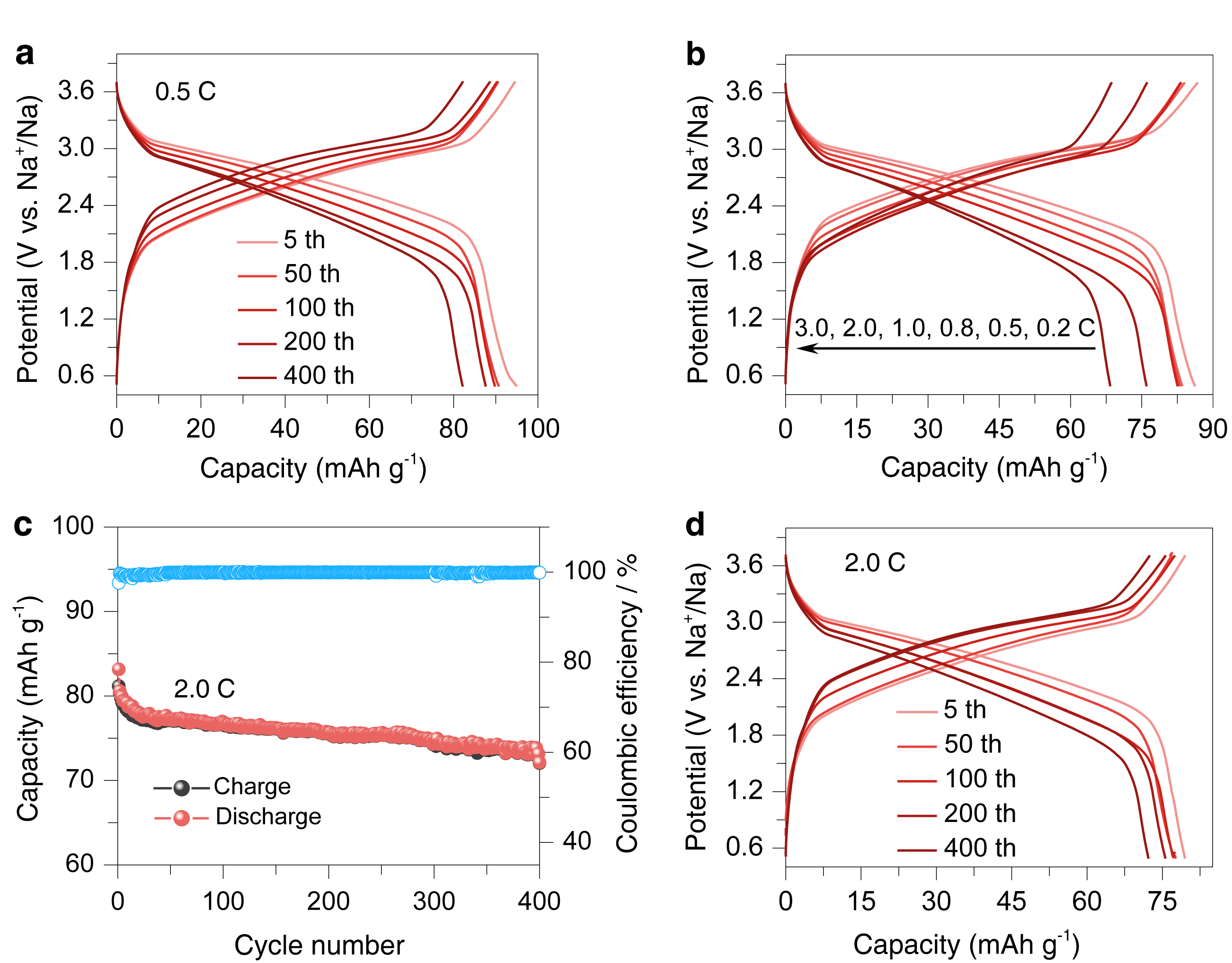


**Fig. S9** Ni_2_P@GPC/CFP||NVP@C full cell performances: **a** voltage-capacity profiles at 0.5 C for selected cycles, **b** rate performance at various C-rates, **c** long-term cycling performance and coulombic efficiency at 2.0 C, and **d** corresponding voltage-capacity profiles at 2.0 C


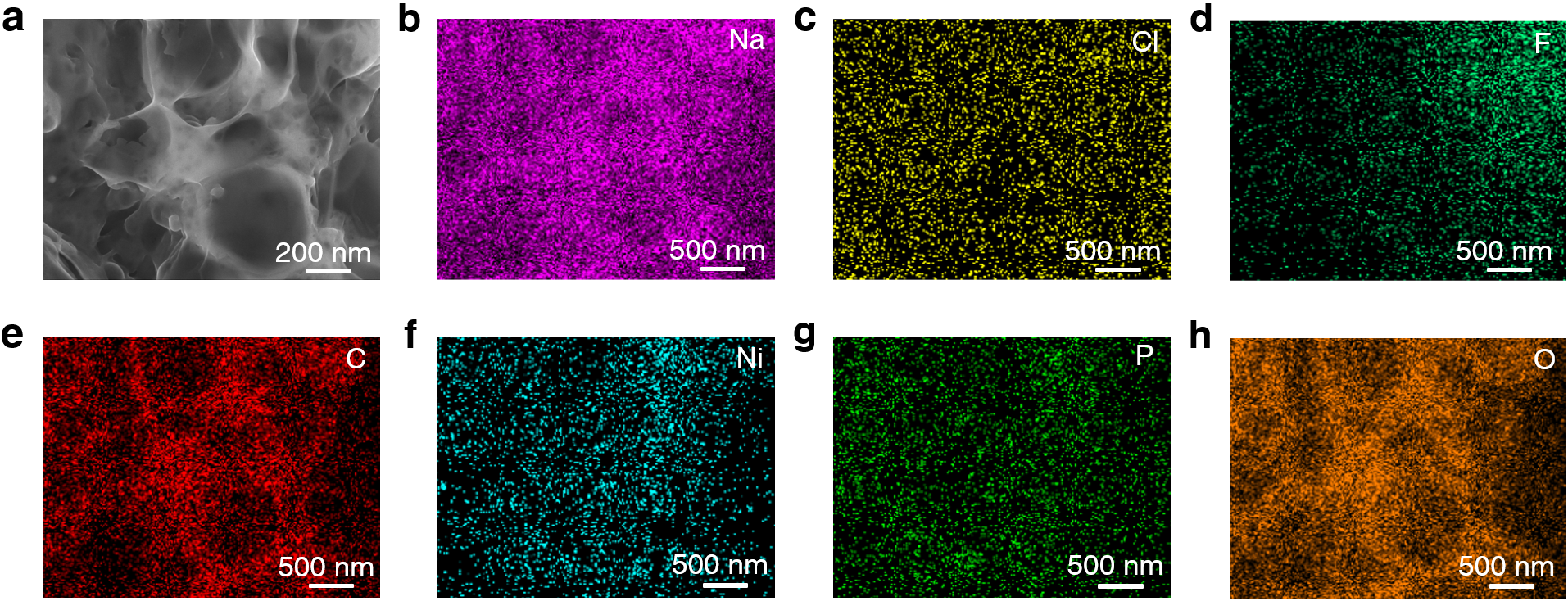


**Fig. S10** **a** SEM image and **b-h** corresponding EDX elemental mappings of Ni_2_P@GPC/CFP after initial cycle at 100 mA g^-1^


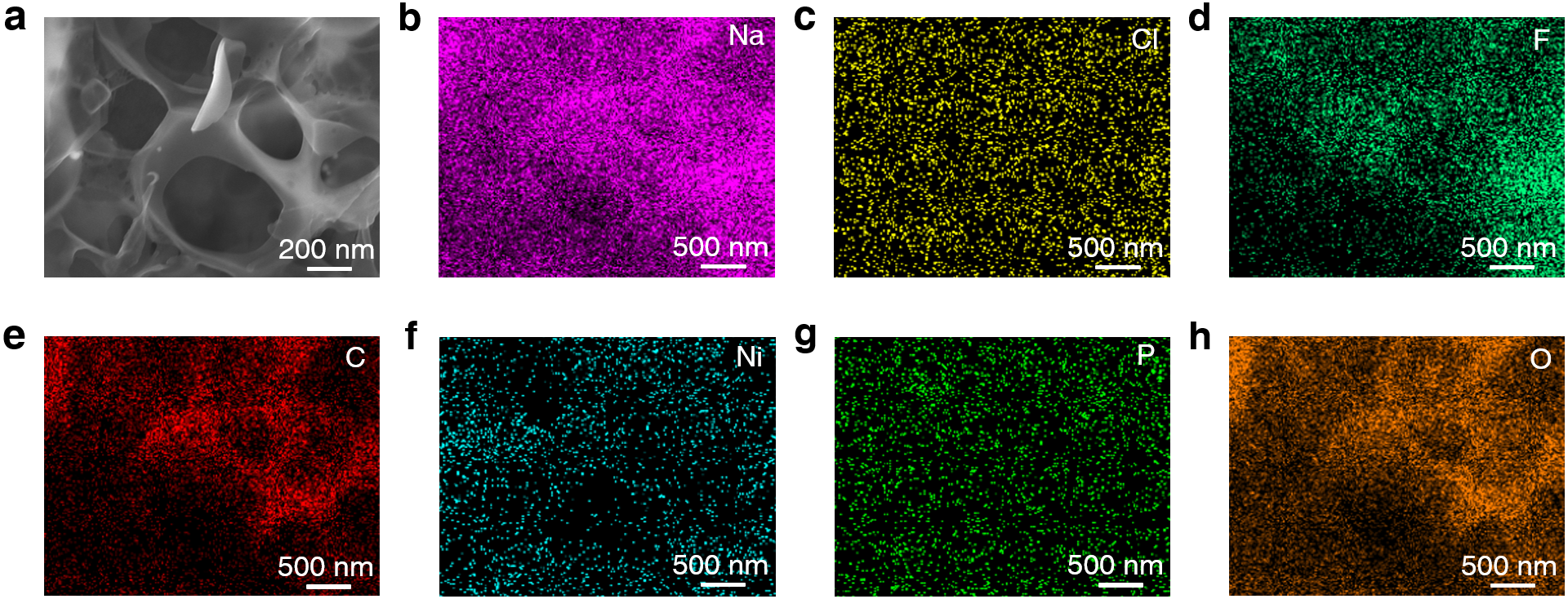


**Fig. S11** **a** SEM image and **b-h** corresponding EDX elemental mappings of Ni_2_P@GPC/CFP after 50 cycles at 100 mA g^-1^


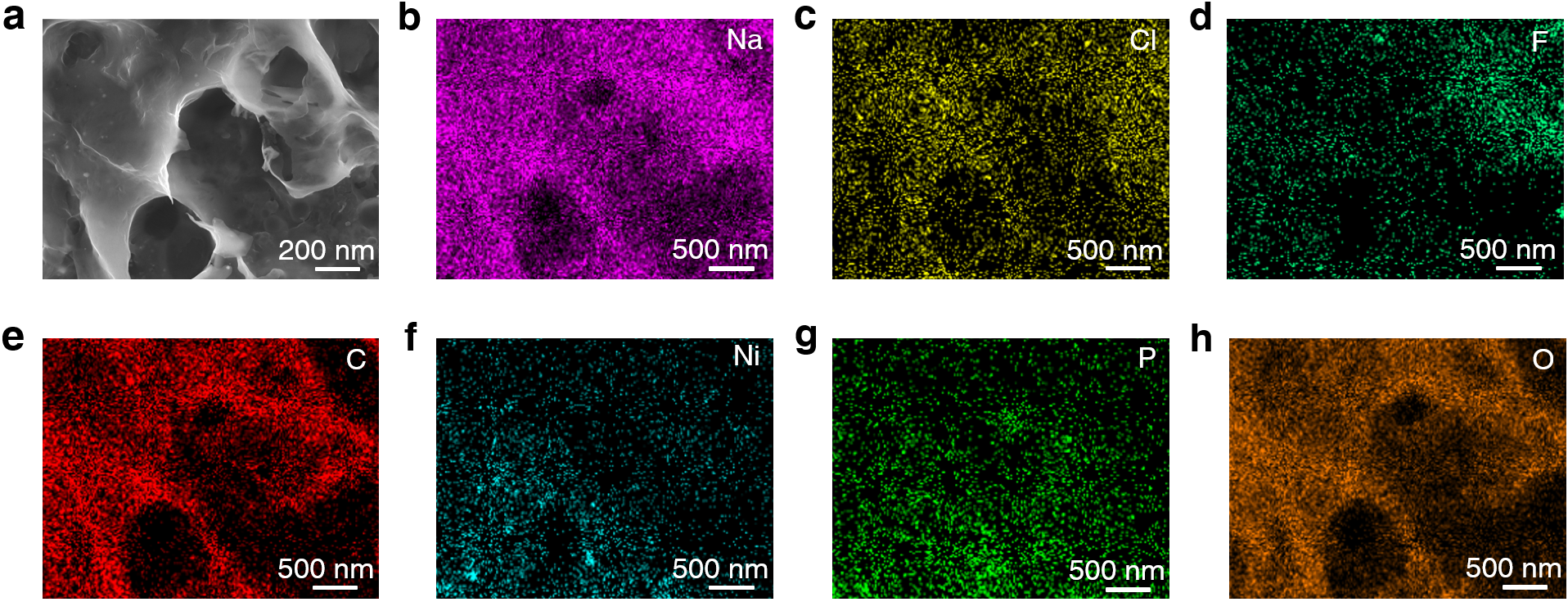


**Fig. S12** **a** SEM image and **b-h** corresponding EDX elemental mappings of Ni_2_P@GPC/CFP after 100 cycles at 100 mA g^-1^


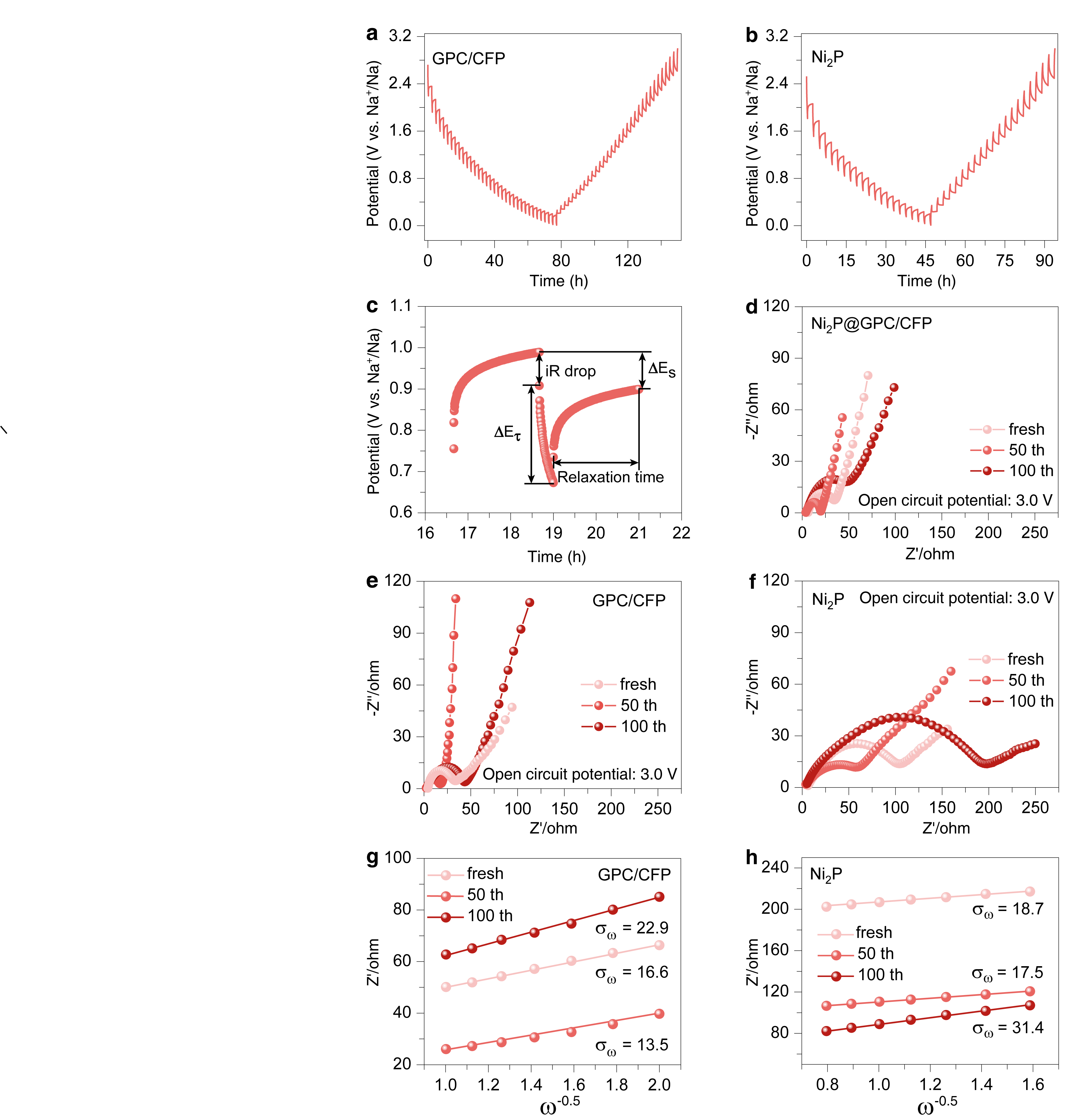


**Fig. S13** **a, b** GITT time-potential distributions of GPC/CFP and Ni_2_P. **c** Illustration of ∆Es, ∆Eτ, and iR drop in GITT. **d-f** Electrochemical impedance spectra (EIS) of Ni_2_P@GPC/CFP, GPC/CFP and Ni_2_P, recorded before cycling and after 50 and 100 cycles. **g-h** Linear fitting of Z′ *vs.* *ω*^-0.5^ for GPC/CFP and Ni_2_P

**Table S2** EIS fitting results and Na^+^ diffusion coefficients ($D_{{Na}^{+}}$) of Ni_2_P@GPC/CFP, GPC/CFP and Ni_2_P in half cells

| Sample | Ni_2_P@GPC/CFP\|\|Na | | | GPC/CFP\|\|Na | | | Ni_2_P\|\|Na | | |
| --- | --- | --- | --- | --- | --- | --- | --- | --- | --- |
|  | R_e_ (Ω) | R_ct_ (Ω) | $D_{{Na}^{+}}$  (cm^2^ s^-1^) | R_e_ (Ω) | R_ct_ (Ω) | $D_{{Na}^{+}}$  (cm^2^ s^-1^) | R_e_ (Ω) | R_ct_ (Ω) | $D_{{Na}^{+}}$  (cm^2^ s^-1^) |
| Fresh | 4.3 | 32.9 | 4.1×10^-10^ | 4.0 | 31.1 | 1.6×10^-10^ | 6.2 | 105.2 | 1.3×10^-10^ |
| 50th cycles | 2.8 | 19.6 | 1.3×10^-9^ | 2.4 | 16.2 | 2.7×10^-10^ | 3.7 | 71.2 | 1.4×10^-10^ |
| 100th cycles | 4.1 | 56.4 | 2.1×10^-10^ | 3.9 | 42 | 8.3×10^-11^ | 5.2 | 189.0 | 4.4×10^-11^ |


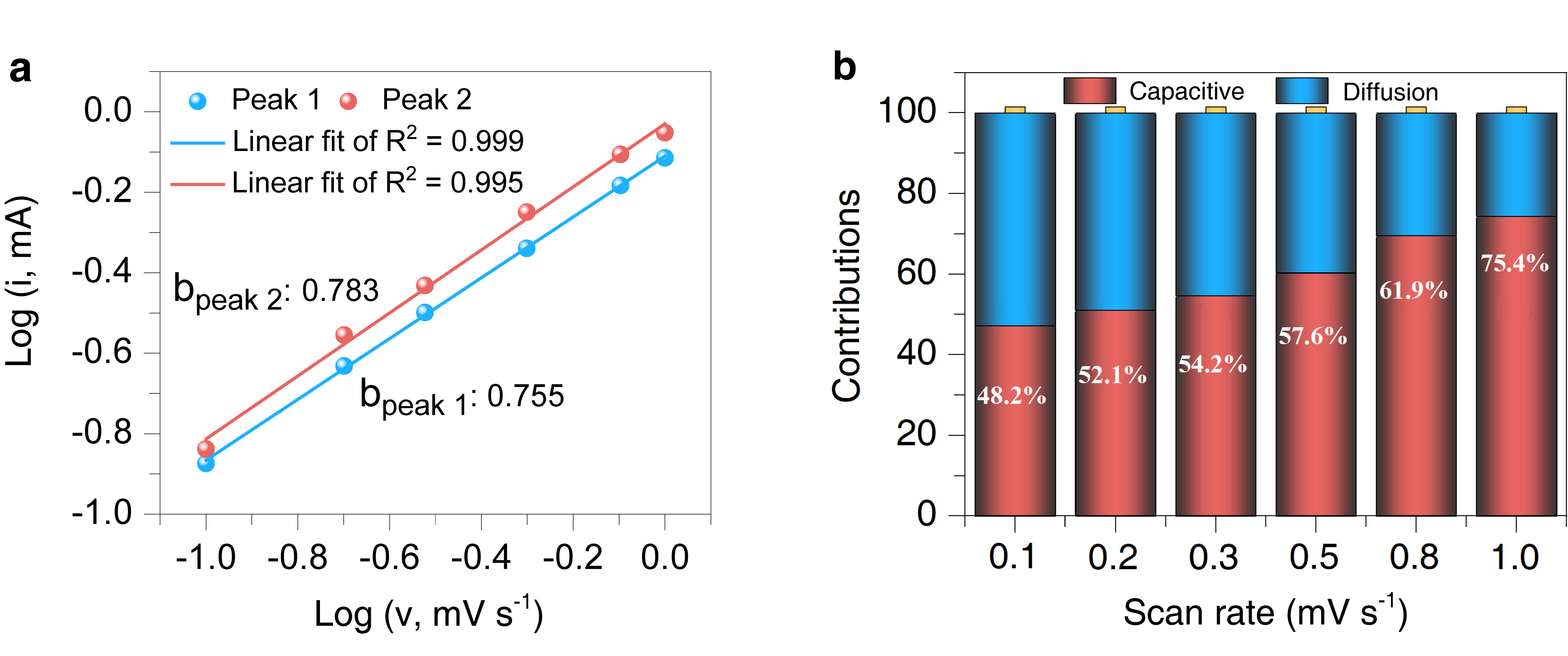


**Fig. S14** **a** b-values determined from the linear fitting of log(i) and log(*v*). **b** Capacitive contribution at different scan rates for Ni_2_P@GPC/CFP


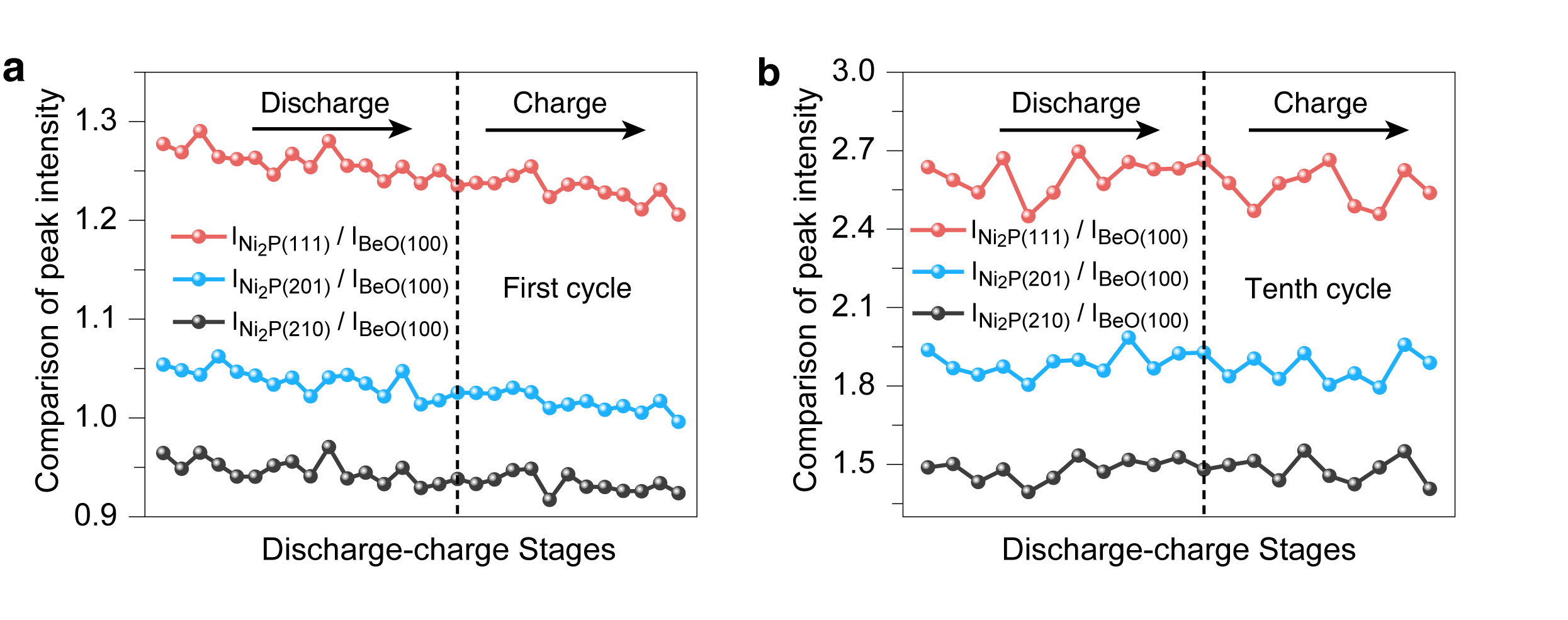


**Fig. S15** Evolution of the peak intensity ratios of Ni_2_P (111), (201), (210) relative to BeO (100) during **a** the first and **b** the tenth cycle


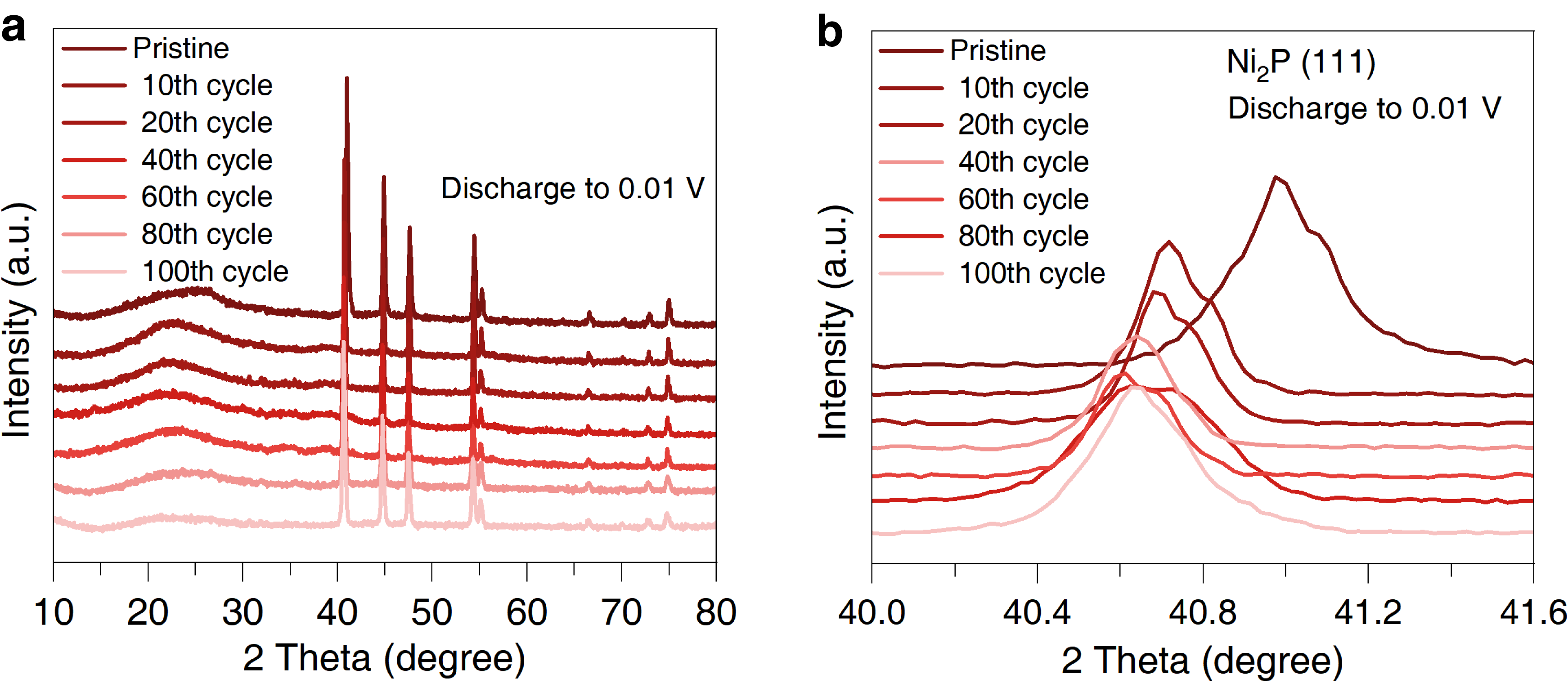


**Fig. S16 a** Ex-situ XRD patterns of the Ni_2_P@GPC/CFP electrode discharged to 0.01 V after different cycles. **b** Enlarged view of the Ni_2_P (111) diffraction peak in the 2θ range of (40°~41.6°)


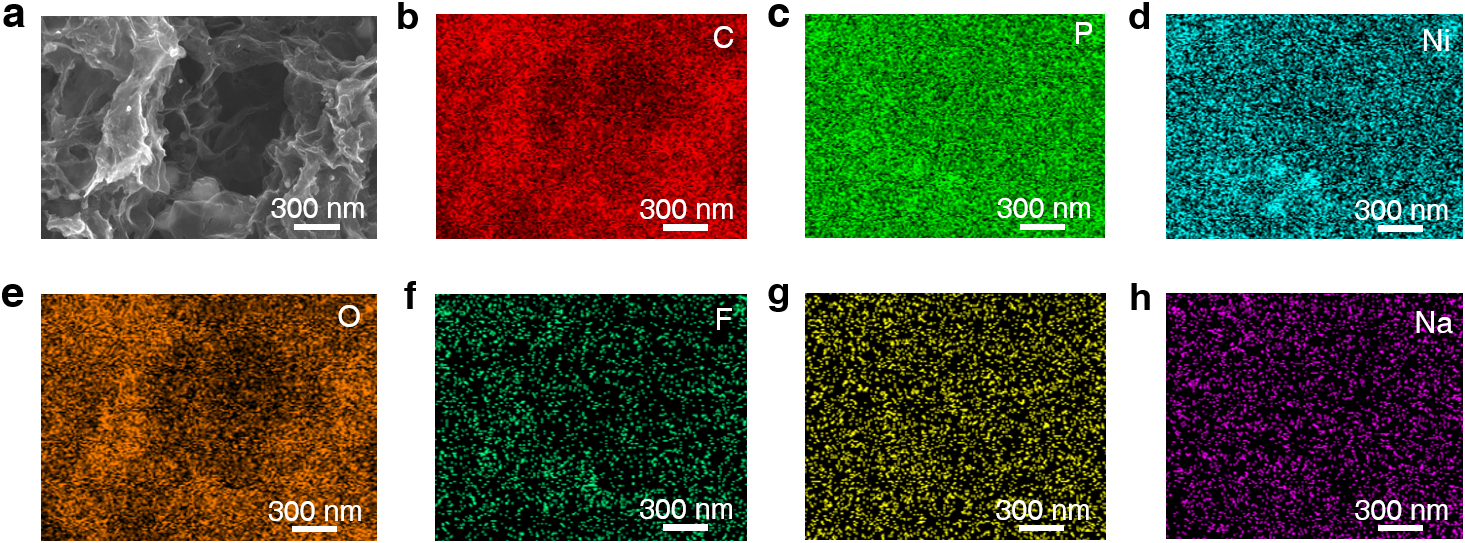


**Fig. S17 a** SEM image and **b-h** corresponding EDX elemental mappings of Ni_2_P@GPC/CFP electrode discharged to 0.01 V after 10 cycles at 100 mA g^-1^


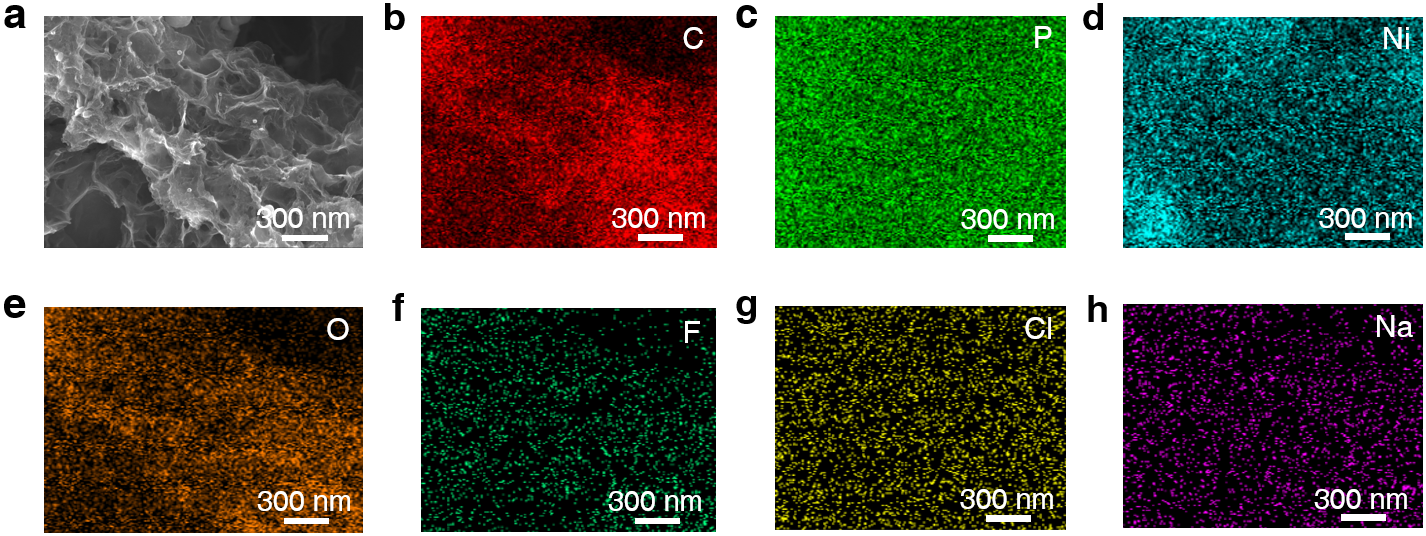


**Fig. S18 a** SEM image and **b-h** corresponding EDX elemental mappings of Ni_2_P@GPC/CFP electrode discharged to 1.2 V after 10 cycles at 100 mA g^-1^


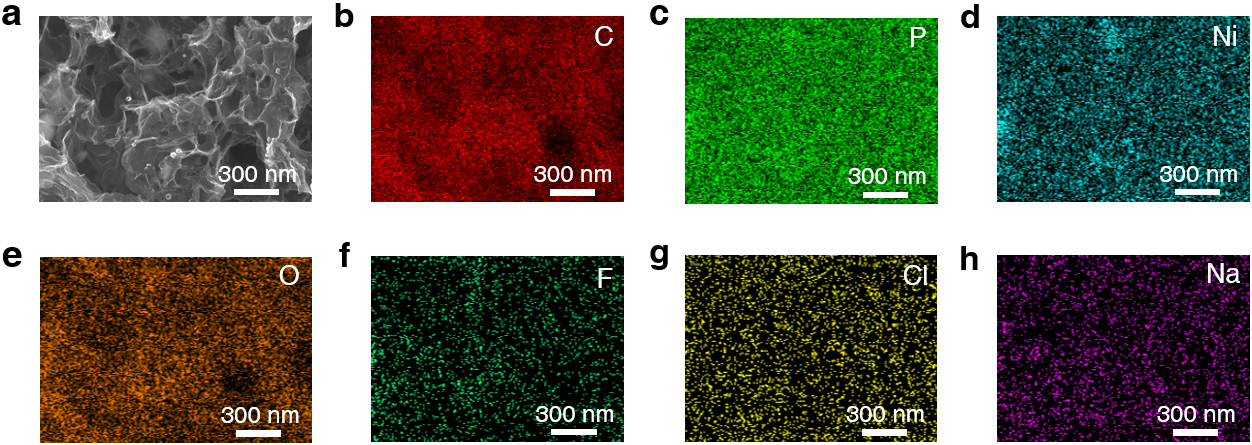


**Fig. S19 a** SEM image and **b-h** corresponding EDX elemental mappings of Ni_2_P@GPC/CFP electrode charged to 3.0 V after 10 cycles at 100 mA g^-1^

**Supplementary References**

1. H. Ou, P. Li, C. Jiang, Y. Liu, Y. Luo et al., Synergistic enhancement of Ni_2_P anode for high lithium/sodium storage by N, P, S triply-doping and soft template-assisted strategy. J. Colloid Interface Sci. **678**, 365-377 (2023). <https://doi.org/10.1016/j.jcis.2024.08.182>
2. S. Liu, Q. Shi, X. Liu, J. Fan, L. Ren et al., Ni_2_P/Cu_3_P bimetallic phosphide embedded in nitrogen-doped carbon as SIB anode: Constructed hetero interface promoting fast Na^+^ diffusion and storage performance. Chem. Eng. J. **502**, 157889 (2024). <https://doi.org/10.1016/j.cej.2024.157889>
3. H. Zhou, Y. Zhao, Y. Jin, Q. Fan, Y. Dong et al., Bimetallic phosphide Ni_2_P/CoP@rGO heterostructure for high-performance lithium/sodium-ion batteries. J. Power Sources **560**, 232715 (2023). <https://doi.org/10.1016/j.jpowsour.2023.232715>
4. B. Cheng, B. Wang, H. Lei, F. Zhang, X. Liu et al., Nickel sulfide/nickel phosphide heterostructures anchored on porous carbon nanosheets with rapid electron/ion transport dynamics for sodium-ion half/full batteries. J. Colloid Interface Sci. **643**, 574-584 (2023). <https://doi.org/10.1016/j.jcis.2023.03.134>
5. S. Liu, Q. Shi, X. Liu, M. Zhang, F. Lin et al., Rational tailoring the hetero-architectures of Ni_2_P/CoP_2_ for stable and high-power sodium-ion batteries. J. Energy Storage **126**, 117026 (2025). <https://doi.org/10.1016/j.est.2025.117026>
6. Z. Luo, S. Liu, Y. Zhao, J. Li, D. Yang et al., Iron phosphides nanocrystals encapsulated with hierarchical nitrogen doped carbon networks as high performance anodes for sodium ion batteries. J. Power Sources **629**, 235972 (2025). <https://doi.org/10.1016/j.jpowsour.2024.235972>
7. Q. Zheng, S. Zhou, S. Tang, H. Zeng, Y. Tang et al., Unveiling atom migration abilities affected anode performance of sodium-ion batteries. Angew. Chem. Int. Ed. **62**, e202303343 (2023). <https://doi.org/10.1002/anie.202303343>
8. Y. Liu, J. Wang, Q. Shi, M. Yan, S. Zhao et al., A stress self-adaptive structure to suppress the chemo-mechanical degradation for high rate and ultralong cycle life sodium ion batteries. Angew. Chem. Int. Ed. **62**, e202303875 (2023). <https://doi.org/10.1002/anie.202303875>
9. J. Jiang, C. Ma, W. Zhang, Y. He, X. Li et al., Controlled design for integration of FeP into 3D carbon frameworks for superior Na storage. Chem. Eng. J. **429**, 132271 (2022). <https://doi.org/10.1016/j.cej.2021.132271>
10. Y. Jiang, M. Xie, F. Wu, Z. Ye, Y. Zhou et al., Metal-organic framework derived cobalt phosphide nanoparticles encapsulated within hierarchical hollow carbon superstructure for stable sodium storage. Chem. Eng. J. **438**, 134279 (2022). <https://doi.org/10.1016/j.cej.2021.134279>
